# Urban Agriculture as an Alternative Source of Food and Water Security in Today’s Sustainable Cities

**DOI:** 10.3390/ijerph192315597

**Published:** 2022-11-24

**Authors:** Aleksandra Nowysz, Łukasz Mazur, Magdalena Daria Vaverková, Eugeniusz Koda, Jan Winkler

**Affiliations:** 1 Department of Revitalization and Architecture, Institute of Civil Engineering, Warsaw University of Life Sciences—SGGW, Nowoursynowska 159, 02-776 Warsaw, Poland; 2 Department of Applied and Landscape Ecology, Faculty of AgriSciences, Mendel University in Brno, Zemědělská 1, 613 00 Brno, Czech Republic; 3 Department of Plant Biology, Faculty of AgriSciences, Mendel University in Brno, Zemědělská 1, 613 00 Brno, Czech Republic

**Keywords:** urban agriculture, urban farm, community garden, allotment garden, circular economy

## Abstract

The concept of a regenerative city goes far beyond a sustainable one. The regenerative approach is to think of urban green space as a productive landscape, a source of food, and a support for biodiversity. In this approach, the so-called urban wastelands have a positive significance. Urban agriculture (UA) has become a commonly discussed topic in recent years with respect to sustainable development. Therefore, the combination of urban fabric and local food production is crucial for ecological reasons. The key issues are the reduction of food miles and the demand for processed food, the production of which strains the natural environment. At the same time, UA enables regeneration and restoration. An original methodological approach was used in the study following the mixed-method research concept: literature survey, case studies, and comparative analysis of objects. A review of UA architecture (UAA) projects was carried out to supplement the knowledge acquired during the bibliometric analysis. In sum, 25 existing projects, including allotment gardens, community gardens, and urban farms in the global north, were compared in this study. As a result of the analyses carried out, the breakdown of urban agriculture was developed into the following categories: (i) architectural–urban, (ii) ecological, (iii) social, and (iv) economic, including the impact of UA on physical activity and social interaction. UA is also a factor shaping the urban landscape. In conclusion, agrarian practice in urban environments has led to the creation of a new type of space, known as UAA. Production in the context of UA exceeds private goods, such as food produced for sale or for individual use. Additional goods include public goods. The review shows that UA fulfills economic, social, and environmental functions, thus falling under the concept of sustainable development.

## 1. Introduction

Since the 1990s, sustainable urban development has been implemented worldwide in response to a range of problems, such as urban sprawl, pollution, traffic congestion, economic decline in developed countries, and rapid urbanization in developing countries [1].The concept of sustainability has been widely discussed by various disciplines over the last and current century [1,2,3,4,5,6].The aim was to raise environmental awareness about the use of natural resources in cities [7]. Thirty years later, some researchers argue whether the sustainability paradigm is sufficient in the face of the climate crisis we are facing and which effects we are already experiencing [8,9,10,11,12,13] Therefore, this paradigm is criticized from a perspective that points to global inequalities in development and the progressive scarcity of natural resources. This criticism has led to the search for even more progressive ways of thinking about urban “development,” which are defined by notions such as circular economy (CE) [14,15,16,17,18], urban resilience [19,20,21], urban metabolism (UM) [22,23], nature-based solutions [6,24,25] and ecological renewal [26], but also degrowth (Oslo Architectur Triennale, 2019). These concepts point to the formation of a regenerative city paradigm that goes far beyond a sustainable one. Therefore, it might be more useful in the context of the current crises: environmental, social, and economic.

The CE concept aims to eliminate the waste of materials by bringing them back into second use. Looping natural resources in the life cycle reduces the need to extract natural resources. The main goal of CE is the harmonious development of humanity and the introduction of sustainability elements into the economy without adverse impact on the existing ecosystem [27,28]. CE is sustainable but also regenerative, considering composting of organic waste and soil production. Therefore, it is important to implement CE in urban green areas.

An inherent value of open green spaces in an urban environment is, among others, urban ventilation, reducing temperatures (alleviate heat island problems), absorbing carbon dioxide, releasing oxygen, water retention, and enhancing biodiversity [29,30,31,32]. In addition to urban greenery (green infrastructure), water plays a key ecological and social role in the cities (blue infrastructure). It brings us to the topic of water management, including the issue of water reuse in the context of CE.

Another approach is to think of urban green space as a productive landscape, a source of food and a support for biodiversity. In this approach, the so-called urban wastelands have a positive significance. Urban agriculture (UA) has become a commonly discussed topic in recent years with respect to sustainable development [33,34,35]. Therefore, the combination of urban fabric and local food production is crucial for ecological reasons [36,37,38,39,40,41]. The key issues are the reduction of food miles and the demand for processed food, production of which strains the natural environment [42,43,44]. At the same time, UA enables regeneration and restoration. Furthermore, community gardens and urban farms are living laboratories of CE [16,18]. The current interest in UA is related to the growing social awareness in developed countries, which is manifested in the desire to produce and consume local and good quality food, as well as to live in the vicinity of greenery [45]. Research on sustainable and regenerative UA is connected with the issue of water use for cultivation. Reuse of polluted water and rainwater subjected to treatment processes can be combined to supply urban farms with water [46,47].

As mentioned, an aspect is urban food production, of which many grassroots examples were naturally developed according to CE. These are components of alternative food networks (AFN) [48], which contribute to food systems. The purpose of this work is to review such projects. The focus of this paper is urban agriculture architecture (UAA), developed during urban architecture design or of vernacular nature. To recognize global trends in urban agriculture, 25 projects have been compared. They are characterized by low or medium production levels and occur in urban centers or on the outskirts of various cities in the world. The paper discusses over 10 of the selected UAA examples. The work aims at determining the benefits, restrictions, and problems related to designing the agricultural function in an urban environment.

## 2. Materials and Methods

An original methodological approach was used in the study, following the mixed-method research concept: literature survey, case studies, and comparative analysis of objects. A review of UAA projects was carried out to supplement the knowledge acquired during the bibliometric analysis. A total of 25 existing projects, categorized as allotment gardens, community gardens, and urban farms in the global north, were compared in this study. The scope of the research is presented in Table 1. Case selection was done based on researcher knowledge and experience, and the most representative cases were selected for each type in order to depict the current trends in, taking into account different historical, social and political contexts.

This section is divided by subsections providing a concise and precise description of the experimental results, their interpretation, and the experimental conclusions that can be drawn.

The survey includes: (i) understanding of the structure and morphology of the case, and occurring processes, (ii) elaborating its characteristics, basic values and its functioning and development, (iii) analyses of case location and characteristic features of the location (context, functional zone, space type), (iv) mutual relationships of urban green zones with development areas, and (v) organization model (type of producers and production aim). Furthermore, ways of negotiating territoriality and its influence on publicness are examined as a social sustainability indicator. The following techniques were used: (i) documentary study (architectural and urban planning projects, documentation prepared by entities managing and using the object); (ii) morphological study (map analysis, orthophotography, systematic photographic survey); (iii) walking survey, expert on-site assessment; and nonparticipant observation. The conclusions are drawn based on specialist publications in gardening and websites managed by community gardens and city farm founding groups, as well as allotment garden boards. The results were compiled in the form of descriptions, illustrations, cumulative maps, tables, and diagrams.

## 3. Allotment Gardens

Allotment gardens originated in Europe in the 19th century as a result of the industrial revolution. Industrialization increased the speed of urbanization processes due to the massive inflow of rural populations into cities. In consequence, the UK and Germany were the first countries to encounter the problem of massive internal migration and expansion of poor quarters [49] in cities. Due to the large scale of these processes, part of the rural population with knowledge and agricultural experience that could have been used for farming edible plants for their own needs did not find employment in cities. Thus, the first allotment gardens were developed for the unemployed to produce food for their families [50].

**Figure 1:**
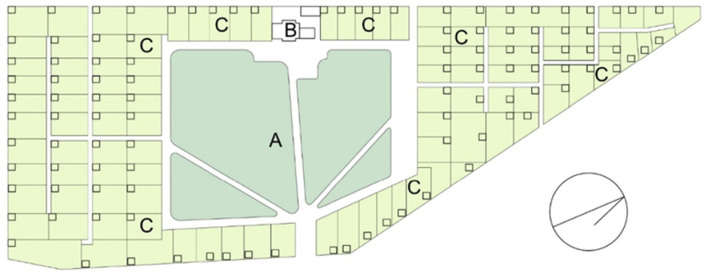
Scheme of the garden colony *Schrebergärten* in Leipzig (Germany), 1909. A—recreational square, B—allotment board building, C—garden lots, each with a summerhouse (compiled by A. Nowysz, original study, based on local visit).

**Figure 2:**
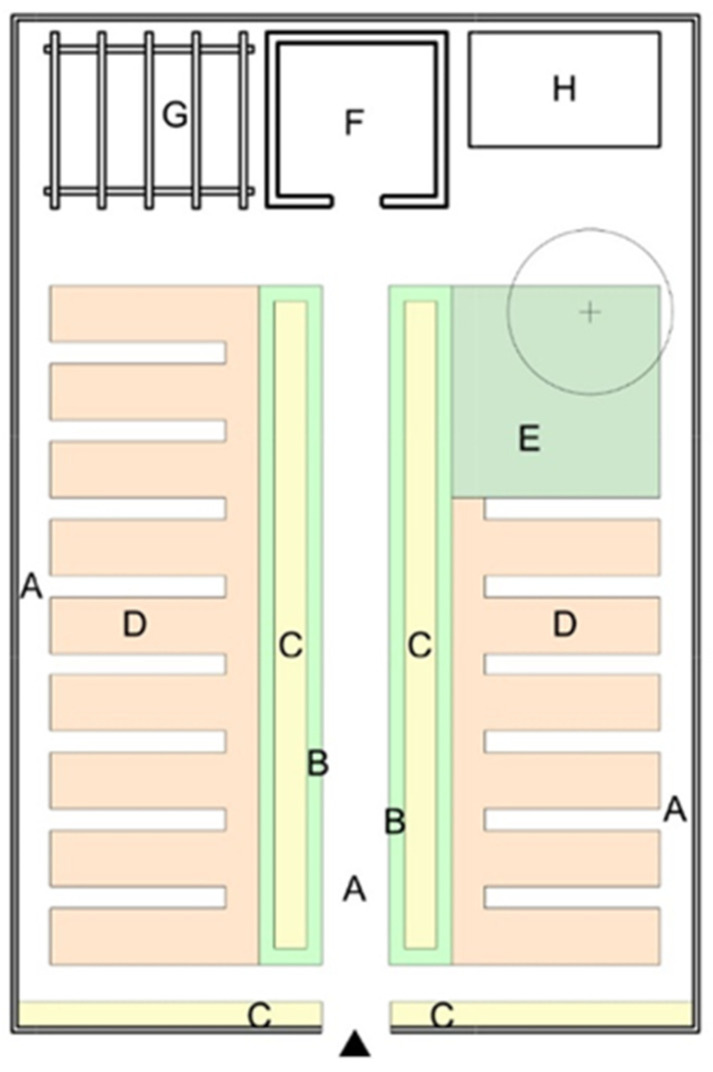
Scheme of a typical lot in the Schrebergärten garden colony in Leipzig, 1909. A—paths, B—decoration hedge, C—decoration flowers, D—edible plants, E—fruit trees, F—summerhouse, G—terrace with construction for grapevine plants, H—composter (compiled by A. Nowysz, original study, based on local visit).

When analyzing the evolution of allotment gardens, it can be stated that the concept of smallholder space in cities (including allotment gardens), developed in the UK, was later rationalized and included in modernistic design in Germany [49]. Allotment gardens were created as individual colonies or as parts of housing estates, near workplaces and schools. The Schrebergärten with a central recreation square belong to the most common allotment establishments in Germany and have been successfully applied also in other countries. A model example of a Schrebergärten is the garden established in 1868 in Leipzig [51]. At first, the garden consisted only of a recreation square, because it did not have an agricultural function at that time. The name “Schrebergärten” comes from Mortiz Schreber, who propagated the idea of outdoor educational activities for children and youth in Germany (Supplementary Materials). In turn, Heinrich Gessel, the educator, introduced the concept of surrounding the recreation square with allotment gardens and engaging children in growing plants. Additionally, theatre workshops and handicraft activities, music lessons, hiking, festivals, and lectures on healthy lifestyles and bringing up children were organized in the garden in Leipzig; it became a site for a library, a local newspaper and various social campaigns [52] (Figure 1). At present, the garden colony with its entire infrastructure is under protection, being a display of the Museum of German Allotment Gardens (Deutsches Kleingärtnermuseum) (Figure 2).

Cooperatives and utopian urban discourse developed the allotment movement. In 1898, Ebenzer Howard presented the concept of garden cities, in which as in a lens he concentrated the contemporary dream of an ideal city, established and managed by a cooperative, independent with regard to food requirements, and full of green recreational zones [53]. As a result, several garden settlements for laborers were established, particularly between the world wars. In these modernistic settlements, housing is entwined with usable green zones in the form of allotment gardens [54].

**Figure 3:**
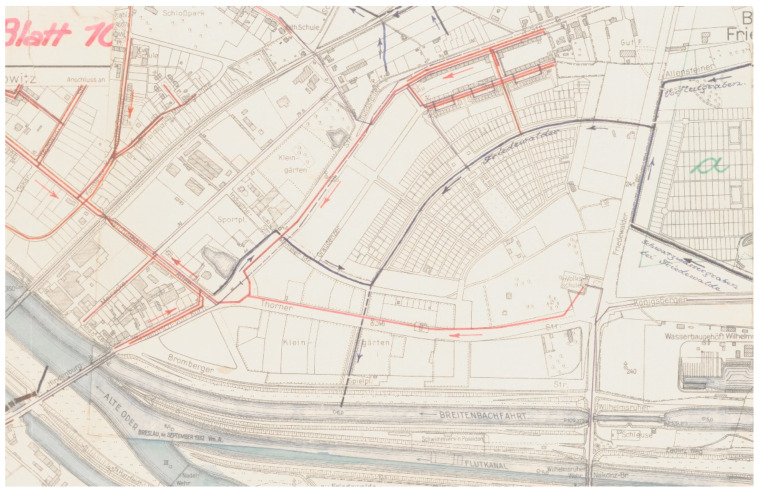
Fragment of section plan of Wrocław (at that time Ger. Breslau) from the 1930, with an establishment comprising an allotment garden colony—at present “ROD Wytchnienie” (Reproduction of archival documents from the collections of the Building Archive in Wrocław, sign. 110479).

A good example is the still existing colony of allotment gardens ROD Wytchnienie in Wrocław, which is part of an antique pattern of a garden establishment, based on a design from 1930 by the German architects Theo Effenberger and Hans Thomas [55]. The establishment has an arched shape of a “fan,” with low modernistic residential buildings in the north and gardens (ROD Wytchnienie) in the south. The southernmost frontage borders with the allotment gardens, and thus the houses are directly connected with them. The longer axis is marked by the main pedestrian avenue; lined on both sides with linden trees and with a narrow channel in the middle, it is the main element of this composition. Moreover, the pattern of the garden colony is wedge-shaped: the area is transversely subdivided by four open-access pedestrian avenues, from which closed-access paths lead to particular lots. A square with a presently unused basin hollow is located in the center of the composition, where the middle transverse and longitudinal avenues cross. This pattern with a central square is an expansion of the Schreber model. The ROD Wytchnienie garden was meant to be a recreational zone for residential buildings, and this is the role it also plays today. The composition of the establishment has remained almost intact since 1945, connected with the garden city idea [55] (Figure 3).

**Figure 4:**
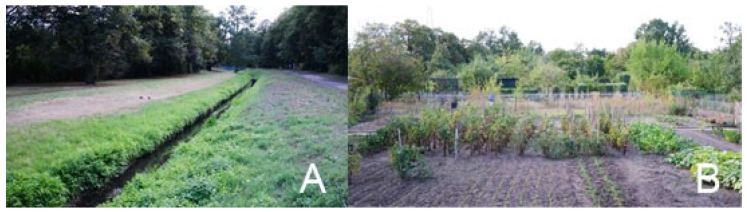
(**A**) Open-access strolling avenue with a channel in the center of ROD Wytchnienie [photo by. A. Nowysz—site visit (06.2017)].); (**B**) an individual lot with cultivation of edible plants in ROD Wytchnienie [photo by. A. Nowysz—site visit (06.2017)].

The allotment colony was designed to combine the agricultural function with open-access strolling grounds (Figure 4A). In order to create a landscape of an urban park, planting of selected fruit trees and bushes was designed on individually lent lots (ROD Wytchnienie, 2021) (Figure 4B).

The gardens described above are just two historical model examples of allotment colonies, which are currently also used by the local community. Recently, in the context of urban development design, allotment gardens play a number of ecological–social functions. They are urban green zones, regions of the activities of residents-tenants, and integration sites for the local community [56]. Similarly, as in other urban green zones, these gardens are used for urban ventilation. They are also biologically active zones, habitats of fauna and flora, and play a role in water retention. However, allotment gardens as a type of urban green zone clearly step out of line from conventional town planning and design, because they are shaped by their users, who generally are not professionals, and the style of this space is usually rural in character [56]. Thus, allotment gardens create a vernacular landscape. The lots contain objects built partly or completely by their users, partly from salvage materials. Therefore, each lot is a place of creative expression of its user, an original search for solutions often exceeding the generally accepted and existing aesthetic norms.

The Polish authors of the ethnographic monograph about allotments Dzieło-Działka draw attention to the fact that for their users, gardens are “zones isolated from the rest of the world, tamed and safe” [57]. Allotment garden residents, interviewed during that ethnographic study, emphasized that undertaking garden activities was a turning point in their lives and that these became an important part of their everyday activities [57]. However, a current problem related to allotment gardens points to their exclusive character, reflected in restricted access for people who do not own a lot in a given area. The problem of restricted access to allotment gardens is also linked with various issues: crop ownership (thefts), spatial solutions (building fences), safety (vandalism, robberies), or utilization of the area by people not belonging to the allotment community (strollers, homeless people) [56]. Furthermore, allotment colonies, covering relatively large parts of the city, due to limited access may become spatial barriers. Apart from the possibility of access to individual lots, loaned by individual tenants, closure of large areas within the entire premises and allowing free access only to a limited number of avenues is common practice. Due to these reasons, reference to allotment gardens as public space is controversial, and some researchers deal with this space as semi-private [56,58].

However, public debate over allotment gardens includes the possibility of their partial opening and indicates the benefits of increased access. One of the possible alternatives is the transformation of some allotment gardens into community gardens. Additionally, the parcel form of allotment gardens may become part of a large premise of open-access recreational green space, as in the example discussed above of a settlement with a park and gardens (ROD Wytchnienie). A detailed description of the two selected gardens (Schrebergärten and ROD Wytchnienie) is presented in Supplementary Materials.

## 4. Community Gardens

A community garden represents a local initiative, in which the local community, e.g., neighborhood, is engaged. Community gardens play an important role in urban agriculture and are a valuable resource for urban regeneration [59]. The gardens are managed by a group of community members, usually on a cooperative basis and in partnership with city, district, or institutional (e.g., school) authorities [60]. They differ from allotment gardens in terms of a smaller total surface area and lack of subdivision into particular rental lots. Community gardens are individual or shared plots of land that are managed and operated by members of the local community with limited access to their land to grow fruit, vegetables, and plants and flowers grown for their attractive appearance [59].

Parallel to the development of the allotment movement in Europe, the first community gardens began to be established in North America [61,62,63]. In the United States, already by the end of the nineteenth century vacant lots were made available to residents for food cultivation for their own needs [64]. In 1894, the Pingree’s Potato Patches campaign was conducted in Detroit: vacant lots were made available, tools were provided, and the unemployed were engaged in food tillage [62]. After the success of this concept, similar campaigns were led in other cities of the United States [65], and during the First and Second World Wars, urban gardening, being a food source, became a manifestation of patriotism. Thus, nonresidential areas were used for food production [59,64]. For example, in 1918 in Chicago, in addition to home gardens covering almost 1500 ha, there were also community gardens on 313 ha and school gardens on 80 ha of the city area [66]. These gardens were established on former vacant lots.

In turn, after 1945 community gardens started to be eliminated in the United States, and the interest in urban agriculture decreased significantly. The history of community gardens in the form they exist today began with the counterculture of the 1960s. The reintroduction of urban agriculture into public debate took place in the 1970s, along with the idea of self-sufficiency in the context of energy crisis, rising food prices, and shaping of environmental ethics [62,63,67]. From the end of the 20th century, in Western Europe and the United States this gardening movement has become more common, and at present the users of these gardens assemble in various types of unions: local, national, and international [68] (Bende & Nagy, 2016).

Contemporary communal gardens in the United States usually originate in neglected and vacant lots [67]. Created within regular urban housing, they easily become local centers, effectively contributing to the urban renewal of a given district. This revitalization potential is readily used by local authorities. However, the risk of gentrification is also linked to urban renewal as it happened in some cities [69]). A good example of the use of urban agriculture is a communal activity organized since 2002 in the United States and known as the National Vacant Properties Campaign [70,71]), promoting the use of vacant lots in cities. The motto of this campaign is “Creating opportunity from abandonment” (National Vacant Properties Campaign, 2019). The idea was developed, e.g., in Detroit, where numerous community gardens and city farms were established, and in New York, where community gardens are also created near schools.

The contemporary nongovernmental organization in the United States, Edible School Yard Project, organizes collective organic gardens in school areas. The beginnings of this initiative reach back to 1995, when a school garden was established in the Martin Luther King School in Berkeley, California. Annually, the organization recruits for an educational program, whose part is the creation of a community garden [72]. The Edible School Yard Project is also part of an international platform uniting different institutions working for school gardening: 5510 programs implementing urban agriculture in schools and university have been already registered in its website, among them the interesting Edible School Yard 1 and Edible School Yard 2 solution from New York, designed by the Work Architecture Company (New York, NY, USA) [73].

The Edible Schoolyard projects are aimed at gardening education—expanding school programs with practice in gardening and increase of food consciousness among children and youth: gaining knowledge about tillage, ethical food production and a healthy diet. Equally important is ecological education—organic waste is composted in these gardens, to be later used as natural fertilizers, and water use is minimized by its reuse and rainwater capture in retention basins. Moreover, the Edible Schoolyard projects enrich the recreation space around schools with functional and esthetical functions. The forms of new architectural objects (a communal kitchen and green-house) reflect their internal subdivision into function zones. This formal legibility and applied color on the elevations makes the architecture friendly for its most frequent users—children. School gardens are initiatives of a community comprising pupils, their parents and teachers.

In turn, in Europe, community gardening is the continuation of allotment gardening [74,75]. Most modern German community gardens have been established in a bottom-up approach without the influence of professional architects [76]. Therefore, their architecture falls into the trend of “anarchitecture,” or contemporary vernacularism. In addition, in many cases these gardens have become well-recognizable urban spaces and often fulfill community activities, such as intercultural integration, decrease of discrimination, and prevention of isolation of national minorities [77].In 2014, 80 German institutions signed the manifesto of urban agriculture (Ger. Die Stadt ist unser Garten) to protect the bottom-up creation of community gardens and allotments [78].Examples of German community gardens are the Prinzessinnengärten and Allmende-Kontor in Berlin.

The Prinzessinnengärten is a garden founded on a vacant lot in a residential quarter of the Kreuzberg district [79,80]. Due to polluted urban soil [81], all plants are cultivated in supplied soil in containers, crates, pots, or other salvage vessels. The garden space is reworked on a current basis, but several permanent elements can be distinguished: the main, central avenue, along which market stands are assembled; restaurant zone: bar, kitchen, tables; plant nursery; tillage zone for different vegetables, fruit and herbs; bee yard; recreation zone with a playground for children; storage houses, facilities, and toilets. “anarchitectural” objects can also be found in the garden, designed and built by community members, e.g., restaurant and kitchen made of industrial containers, exhibition stands and small architecture made of Euro-pallets, and unique summerhouses made of various salvage material [78]. The Prinzessinnengärten is an example of a low-budget project of adapting a vacant lot in a residential quarter. As a result, turning a building lot into a tillage lot increases the green area in the dense urban fabric. The space becomes adapted to current requirements. Besides food production, the Prinzessinnengärten is used for social issues, e.g., gardening, food and ecological education, increase of local identity based on work in the local garden, activation and cooperation of various age, cultural, and gender groups, as well as including the residents in the process of urban landscape development. Furthermore, because food production often exceeds self-supply, the garden also gains also significance in an economic aspect, becoming part of the local food system [80].

The next example of a community garden is the Allmende-Kontor located in the former Tempelhof Airport in Berlin, transformed in 2010 into a public park [82,83] 2011, in the eastern part of the former airfield, the Stadtteilgarten Schillerkiez cooperative established the Allmende-Kontor garden. At first a group of 20 people occupied an area of 5000 m^2^, where 10 garden installations made out of reused material, for example, Euro-pallets, crates, or shopping carts, were assembled and filled with soil for vegetable and fruit tillage [84].Further installations were built in the following years and the area of the informal garden gradually expanded.

However, some community gardens are created top-down, following an initiative from the city authorities, cultural or educational institutions. In these cases, the organizational institutions (e.g., local school councils) invite selected architects or artists and future users (e.g., settlement residents, pupils) to cooperate in order to develop collective gardens [59].This model of establishing a community garden fulfils the idea of social participation in shaping the city, in which the active participation of all parties in the design process, decision making, and project achievement is of key importance. In France, numerous community gardens were developed on the basis of projects prepared by architects or artists cooperating with residents of a given settlement—the future gardeners [85,86].

The French studio—Atelier d’Architecture Autogérée (AAA)—by applying the social participation strategy, has accomplished several gardens with various groups of Parisian residents. Architects from AAA refer to their projects as self-managed architecture. The main focus of their projects is a more ecological, democratic and bottom-up managed city [87].The creation of AAA architects exceeds the material object by linking project studies and urban activism. Most activities of AAA are linked with urban agriculture, e.g., the community gardens Ecobox and Passage 56, and the AgroCité farm.

Ecobox is a concept of a mobile community garden, whose location changes depending on the availability of a vacant lot in Paris. The AAA studio has coordinated the establishment of such gardens, e.g., in the La Chapelle district. The first one was created in 2001 on a post-industrial lot near the Halle Pajol complex. The garden had the form of a rectangular platform built of used Euro-pallets in which geometric holes were cut out to serve as patches for plants [88].

Ecobox is thus a project of establishing collective gardens through social participation on vacant lots in Paris. These gardens are created with low financial input and are temporary in character, which influences their “anarchitectural” aesthetics. The main aims of this project are including residents in the process of shaping urban landscape, enhancement of local identity based on work in the local garden, activation and cooperation of various social groups, as well as food security through local production. So far, three such gardens have been established in Paris.

Another example is the Passage 56 community garden, established in 2006 in Paris as a result of the cooperation of the AAA studio and local residents. The project began with public consultation that lasted for several months. Then, the garden developed gradually: the works began in 2006 and 2 years later the area was passed down for maintenance to a group of 40 people [87].

Passage 56 covers a rectangular lot 5 by 40 m in size, which is the empty space between two residential buildings. A two-story wooden installation is located from the side of the street—it is a pavilion whose construction is filled with salvage material. The structure flanks the passage, thus supplementing a break in the frontage, and its lower level plays the role of the garden entrance. The second level of this construction contains the office for the garden members and photovoltaic panels have been installed on its roof. Next, the lot contains a communal-recreational space and patches with tillage. A small construction containing a small storage area, composter and a basin for rainwater is situated in the other end of the garden. Passage 56 is a community garden and the residents have been actively included in the process of creating the urban landscape by participatory design. In effect, a recreational usable garden has been developed, which is maintained by an informal group of the estate residents.

In summary, community aspects and social activities are equally important as food production in collective gardening practice. Furthermore, the open-source concept exists in community gardens, i.e., mutual sharing of knowledge and experience, and common use of space and tools [82]. Beside agricultural functions, these gardens are also places of other activities such as educational courses, thematic workshops, lessons and camps for children and youth, and sports. Moreover, the aims of collective gardening, often determined in the statutes of the community, include shaping social identity, neighborhood integration and allowing individual development of particular community members through social participation [89], engagement, self-organization, activism, exchange of experience and fulfilling various functions (e.g., in the board) [60], as well as architectural practice—the space of many communal gardens is designed and constructed by its users or in cooperation with architects.

Community gardens play a number of social functions, e.g., they are used for ecological and food education, as exemplified by the Edible School Yard school gardens in New York. Furthermore, community gardens, by allowing food production for individual needs, to some extent counteract poverty. Therefore, local governments and nongovernmental organizations readily become engaged in the process of their establishment by: ensuring infrastructure and conducting educational programs and platforms for cooperation, knowledge exchange and information. Such an aim was in the focus of the establishment of many gardens and farms in Detroit (e.g., Lafayette Greens, 2018), from which the produced food is transferred to food banks). Collective gardening is also used in social rehabilitation programs (e.g., in San Francisco [90]) and promotional and reputational activities, e.g., in 2008, a vegetable garden was opened in front of the city hall in San Francisco [91]. A detailed description of the selected community gardens is presented in Supplementary Materials.

## 5. Urban Farms

A city farm is a farmland quarter located in the city. Similarly, as in the case of community gardens, farms are established on vacant lots. They differ in their more productive character, i.e., the farm area is maximally used for the tillage of edible plants, whereas recreation–leisure functions do not occur or seldom occur to a much smaller degree. The war-time vegetable gardens established in Europe and the United States during both world wars represent examples of space corresponding to the description of city farms. However, agricultural space in cities began to be known as city farms as late as in the second half of the twentieth century, e.g., a farm created in the outskirts of London in 1972 [92] and another established in 1984 in London [93].

The basic aim of urban cultivation is supply of fresh fruit and vegetables. Local food production is particularly important in the case of wars or political conflicts or in the case of price speculation for food products. Thus, urban agriculture is one of the elements shaping regional food security [94,95]. Therefore, in some cities, cultivation areas are introduced top-down, e.g., at national or local government level. In this case, urban agriculture is considered as one of the elements forming the local food system, i.e., organization of food production and distribution, as well as space related with its production, distribution, and consumption [47].

**Figure 5:**
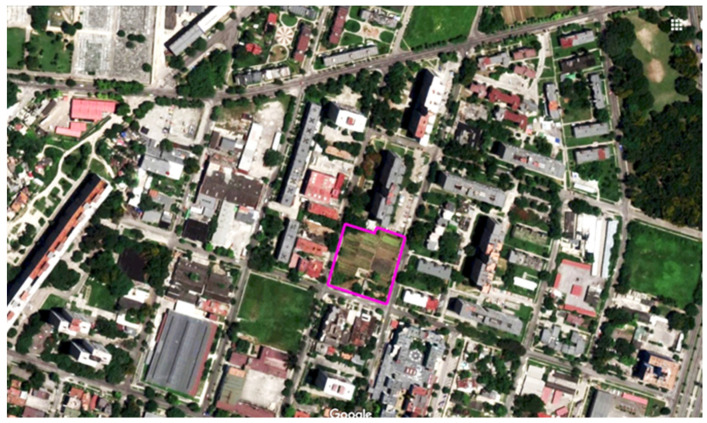
Location of the Organopónico La Sazon farm in Havana (Cuba), (1994) (based on www.google.pl/maps, accessed on 16 August 2018).

Examples of city farms established to ensure the food security of a given area are those in Cuba. The historical background of the phenomenon of urban agriculture in Cuba includes the embargo imposed on the country by the US government in 1962, as well as the economic crisis related with the fall down of the eastern bloc in the 1990s. The difficult economic situation and food deficiency, caused by the rapid loss of trade partners and economic isolation, forced Cubans to work out an alternative economy based on the national internal market instead of export and import. In the 1990s, the Cuban government noticed the economic potential of urban farming and decided to include bottom-up garden initiatives in systemic and organized planning on a central level [96] (Figure 5). The reforms allowed the Cubans to establish small farms for local food production, also in urban and suburban areas. The Department of Urban Agriculture (Spanish Grupo Nacional de Agricultura Urbana) was active in Havana already in 1998 [97].

The example Organopónico La Sazon farm in Havana covers a nonresidential area within a housing estate from the 1980s. The space between the multistory residential buildings remained unused after their construction. A city farm was established here in the 1990s [98]. The farm with a total surface area of about 5.2 ha is subdivided into long patches with a width of about 1.2 m and paths between them with a width of about 0.65 m. The patches are surrounded by 0.2 m high retaining walls built of rocks, bricks, or concrete profiles. The bottom of each patch is also covered with stones, which, by separating the garden soil from the ground, play the role of drainage. Due to possible earlier soil pollution on an urban lot, the plants are grown on supplied soil [99]. The plants are cultivated using low-energy organic methods such as, e.g., permaculture [100]. A trade stand is located at the entrance gate to the Organopónico La Sazon, where vegetables and fruits are sold, supplemented by food grown outside the city. Here, the local residents buy their food and it is also an informal social space where neighborhood interactions take place. Organopónico is a state-owned farm supervised by the Cuban Ministry of Agriculture, and the gardeners employed there have permanent jobs. The spatial pattern of the farm, the dimensions of the patches, the technology and the grown products are also subject to the ministry’s regulations [98].

In other countries, city farms are established also for protecting regional food security—by nonpublic (e.g., nongovernmental organizations) and informal (e.g., cooperatives) organs. Farms created from the bottom up may also be parts of local food systems, as in Detroit, where community gardens and farms have become food sources for poorer residents after the financial crisis in 2007. The MUFI farm is a perfect example of collective farming on the outskirts of Detroit. It was established by the Michigan Urban Farming Initiative (MUFI) and passed down to the local community, that cultivates it for self-supply. Excess food is transferred to food banks [101]. Estate farms also began to be included in the stage of urban-architectural design stage. An example of such a project is Urby Staten Island in New York City. Here, the tillage area is part of the residential area similar to the garden estate in Le Corbusier’s Contemporary City. A farm with a surface area of 1524 m^2^, comprising a greenhouse, composter, community kitchen, and dining room, is located in one part of the courtyard [102]. Additionally, a bee yard with beehives is located on the roof of the object. The farm and bee yard are maintained by a hired person, who, beside a salary, has access to an apartment in the estate. The crops are used by residents and the coffeehouse located on the ground level of the building, while excess products are sold [103].

A European example of a city farm designed as an element of the local food system is the AgroCité in Paris. This farm was established in 2010–2014 in the frame of the R-Urban project by the architectural studio Atelier d’Architecture Autogérée (AAA) mentioned above. The project was focused on strategies of cooperation between bottom-up initiatives related to ecological and urban agriculture. In the description of the R-Urban concept, the architects quote the words of the philosopher André Gorz: “produce what we consume and consume what we produce” [104]. This objective triggered the R-Urban project, in which possibilities of creating closed production-consumer cycles in the city, recycling practices, self-organization of the local community, and collective housing were analyzed. Finally, two objects were accomplished in Paris—the AgroCité farm and the recycling education spot RecyLab [104].

Furthermore, numerous architectural projects of urban farms have developed in the last 20 years, they have been presented in various architectural festivals, such as the Biennale of Architecture and Urbanism in Shenzhen and Expo in Shanghai. They include: Landgrab City, Value Farm, Houtan Park and Floating Fields. These projects were conceptual in character, thus beside new organization of agrarian space the new concepts became triggers for a general debate about urban agriculture and contemporary methods of food production. The experimental project of the “city-farm” district in Almere, the Netherlands, also commenced [105]. A detailed description of the selected urban farms is presented in Supplementary Materials.

## 6. Discussion

Developing architectural concepts and community initiatives point to an increased interest in urban agriculture [59,106]. Among the ideas presented in the present work, the oldest type of urban agricultural space is the allotment garden, which as a form of the urban green zone is commonly included in spatial development design [107,108]. While allotment colonies are usually designed by architects, the arrangement of particular lots remains in the hands of their users, which causes some objects of small architecture to be vernacular in character. Allotment gardens develop as independent colonies, e.g., the Schrebergärten in Leipzig, or are part of a park or an estate development as the ROD Wytchnienie. Both examples of garden developments were established before the Second World War and are used to this day.

The next type of UAA is the community garden, usually established by an organized group on a vacant lot in a residential quarter or in an open area such as a park. The community garden is subdivided into functional zones, such as agrarian and recreational, or has an open form—with free-standing garden containers, pots, and other objects for plant cultivation. Small architecture in community gardens is usually made of salvage material and thus represents an “anarchitectural” trend. The gardens are developed entirely by their users or in partial cooperation with architects, who coordinate the design process based on social participation. Under the concept of community gardens lie cooperative culture, urban activism and ecological movements. In addition, these gardens are examples of alternative methods, originally independent of all official institutions methods of managing public space [46].

In summary, allotment and community gardens are territories in which the residents individually shape the landscape, independently of urban administration. Landscapes result from work—they reflect the engagement of their users [54]. On the other hand, community gardens were never included in urban planning. They are established in vacant lots following the bottom-up activities of the residents or the cooperation of designers, institutions, and the local community. Therefore, they are more temporary than allotments, which also influences their aesthetics and spatial solutions [109].

**Figure 6:**
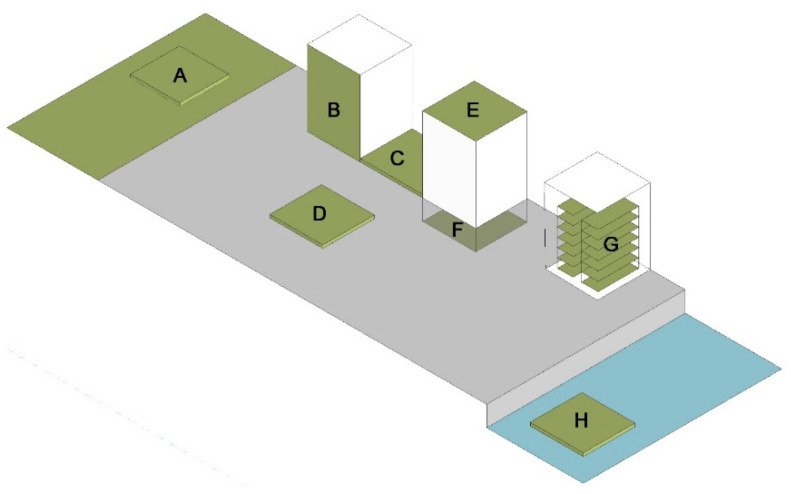
Scheme of the location of the agrarian function in cities. A—nonresidential area (e.g., park), B—building frontage, C—lot between buildings, D—city square, E—building roof, G—building interior, H—reservoir (compiled by A. Nowysz, original study).

A farm is the next type of agrarian space, located in the city between residential areas and parks, on water or roof tops. Farm designs are optimized in terms of productivity: most surfaces are intended for tillage, whereas service and recreational functions are minimal or do not occur at all. Locating farms in cities develops food security, as in the case of the Organopónicos in Havana and collective farms, e.g., AgroCité in Paris and MUFI in Detroit. The farms may be commercial, food being produced for sale, or social in character, food being produced for self-supply or transfer to food banks. In conclusion, the UAA typology distinguishes gardens (community or allotment) and farms. Gardens differ from farms in providing agrarian and recreational functions. In turn, farms are more productive in character. Due to the heterogenic character of the urban landscape, the location of agricultural functions in cities plays a key role. In addition, the locations of the agricultural function in cities (Figure 6) can be characterized, taking into account the urban context, the functional zone and the character of the space (Supplementary Materials).

The next factor distinguishing particular examples is the character of the parties involved in food production in the city. The broadest classification includes the basic subdivision into three types of units [110,111]: state (e.g., local governments); nonstate (e.g., non-governmental organizations); informal (e.g., cooperatives of agricultural producers). The character of the party using a given area determines the production object and the aim (self-supply or market production) (Supplementary Materials). Communal urban farms, community and allotment gardens are of interest to the food security discussion, as they are venues where private and public responses to issues of food insecurity intersect.

## 7. Results

The implementation of urban agriculture has an architectural–urban potential with regard to: (i) functional use of green zones; (ii) use of vacant lots in the urban fabric; (iii) functional enrichment of public space; (iv) increase of green zones in a dense urban fabric; (v) revitalization of postindustrial areas; (vi) development of vernacularism; and (vii) decorative functions. Additionally, depending on the accepted spatial solutions, production technology and management model, urban agriculture may be treated in terms of (1) ecology, (2) society, and (3) economy:

1. In the ecological aspect, urban agriculture is significant due to:
  - biodiversity protection in the urban environment (an appropriate selection of the cultivated plants): habitat for fauna and flora, green zones as ecological rings and corridors;
  - water retention;
  - aeration and enhancement of air quality;
  - composting of organic waste, usage of compost as a natural fertilizer;
  - possible water usage control—usage of grey water and rainwater;
  - possible application of alternative energy sources (e.g., biogas);
  - decreasing demand for processed food, whose production strains the natural environment.
2. In social aspects, urban agriculture is significant due to:
  - food security (broader access to fruit and vegetables);
  - increase of food consciousness: gaining knowledge on tillage, ethical food production, healthy diets;
  - gardening education (e.g., by expanding kindergarten and school lessons with garden activities) and possibility of using the gained skills in the work market;
  - possibility of integrating socially excluded and discriminated groups (e.g., ethnical or national minorities, economically excluded people, elderly);
  - enhancement of the physical and psychical condition of the individuals—users;
  - social participation and inclusion of residents in the process of urban landscape development;
  - enhancement of local identity based on work in a local garden or farm;
  - activation and cooperation of various age, cultural and sex groups;
3. In turn, in an economic aspect, urban agriculture is important due to:
  - local food sovereignty;
  - creating local networks of food producers;
  - reducing food transportation and storage costs as a result of local production;
  - supply of local, seasonal products to small shops and open-air markets;
  - creating new jobs;
  - possibility of obtaining additional income;
  - lower food import;
  - in case of organic waste compositing—possibility of decreasing the local costs of communal waste management;
  - exploitation of unused urban resources such as grey water and rainwater, as well as vacant lots and roofs;
  - establishing new enterprises functioning on the basis of a cooperative model, being an alternative for food monopoly and oligopoly;
  - diversification of economic institutions—supporting the alternative food networks;
  - creating short supply chains

In turn, the restrictions and problems related with urban agriculture include such issues as:

- spatial management policies not taking into account agrarian functions;
- legal regulations prohibiting food production in cities;
- restricted access to arable soil;
- lack of ensuring a stable lease or high cost of urban ground (competition of investors with larger assets, preferring investments ensuring a larger commercial income);
- lack of informal catering and sale of food products to official sanitary requirements;
- heavy metal pollution of soil, water and atmosphere in the former industrial environment;
- occurrence of pathogens;
- gardening practice with negative influence on the natural environment, e.g., grass burning, usage of agrochemicals [112];
- in in-door production: high energy demand of artificial light technology, watering, ventilation, etc.;
- crop or infrastructure vandalism and theft (e.g., in allotment gardens);
- gentrification risk related with revitalization of urban space;
- in a commercial aspect: price competition of imported food.

The safety of food produced in metropolises, similarly as in rural areas, depends on the applied agrarian practice and environmental cleanliness [113]. The quality of food produced in an urban area may be increased through:

- appropriate tillage location—by determining special zones and implementing special regulations;
- monitoring food quality, e.g., by controlling heavy metal contents and pathogen occurrences;
- control and prevention of heavy metal pollution in the urban environment;
- monitoring of liquid and solid waste utilization;
- conducting in-door tillage in a soil-free technology with controlled microclimate;
- food education of producers and consumers.

## 8. Conclusions

The idea of food production in cities for individual needs seems in striking contrast with the character of urban life. It is usually associated with life in the country, with different neighborhood interactions and economic relationships [77]. However, the phenomenon of urban agriculture, including food production for individual needs is a fact and the cultivation of edible plants is one of the activities undertaken by city residents. The aim of such actions may be social or gardening activities, but in many cases plant cultivation has an economic base and is the source of food for poor and socially excluded people. On one hand, urban farming is part of a wider movement—socializing the urban space and self-organization of its inhabitants, but on the other hand it may be commercial in character, as well as be part of a complex food system, influencing regional food security [33,113].

UA is also a factor shaping the urban landscape [114]. An example of the influence of farming on the city structure is including the agrarian function in remediation projects, as well as designing farmlands in the city outskirts, which may contribute to restricting urban sprawl. The basic urban distinction is subdivision into projects in which the agrarian function is one of the elements determining city organization and those in which it has been introduced in the existing urban space (built up or vacant). The first case is more holistic in character and due to its scale usually occurs in theoretic projects, e.g., the garden city of Ebenezer Howard. This article focuses on the second type and presents examples of creating UAA through implementation of agrarian plants in the existing urban fabric.

The agrarian function may occur both in built-up and open areas, forming green zones. In built up areas, urban agriculture is situated both in downtown and suburban contexts, in urban space, e.g., by covering a vacant lot in a residential quarter, and in architectural space, e.g., in building interiors, on roofs or elevations. Urban agriculture is situated in different functional zones, particularly in housing estates, postindustrial areas, service and recreational zones. The agrarian function is also present in the public space, enriching its functions. Furthermore, location of agriculture within city limits is an economically attractive solution due to direct consumers and existence of an organized market.

Edible plants are grown, mainly vegetables, fruit and herbs, as well as decoration plants. In some cases, small-scale animal farming has also been observed, e.g., bee yards, poultry and fish farming. The product depends on the type and subject of production. The basic aim of production in allotment and community gardens is self-supply, whereas farms are commercial in character or serve as self-supply in the case of collective estate farms.

Various attempts of UA management are undertaken in order to revitalize the degraded space, as well as support the fight against poverty and build up regional food sovereignty. Foundations are established to support the organization of food systems, which develop platforms for communication and cooperation of various parties linked with food production, storage and distribution, and waste composting.

Moreover, UA as a part of blue–green infrastructure can supply current needs and contribute to food and water security. Currently, UA is not a complete replacement for rural agriculture. This is impossible because of the excessive demand for food globally, including the problem of hunger. Moreover, urban farming is limited by access to land and water, which also determines the choice of plant species. Nevertheless, diversification and decentralization of food production are crucial for food security. Therefore, UA is only relevant as an element of the overall food system, however, it strengthens its resilience. The role of integration between urban edible green and blue infrastructure occurs in rain gardens and retention basins, which are common for UAA [46]. In addition, UA can play a relevant role in the long term in shaping and transforming global food systems, particularly supporting the alternative food networks. The ecological rationale for UA stems from the possibility to partial transition from extensive agriculture and start the process of degraded rural area reclamation.

To conclude, agrarian practice in urban environments has led to shaping a new type of space, known as urban agriculture architecture. Production in the frame of urban agriculture exceeds private goods, such as food produced for sale or for individual use. Additional goods include public ones. The presented examples show that urban agriculture fulfils economic, social and environmental functions, and thus falls under the concept of sustainable development. Moreover, urban agriculture may be used to revitalize degraded areas and become an actual backup in crisis situations, supporting regional food sovereignty. All mentioned aspects constitute an ecosystem, or a biologically active infrastructure that may be seen as the implementation of sustainable or even regenerative concept [115]. Compared to the concept of sustainable development, the regenerative approach aims not only to reduce the effects of social, ecological, and economic challenges but also to transform them into regenerative change [116,117]. The main challenge is to focus now on developing sustainable and resilient local food systems.

## Figures and Tables

**Table 1:** List of 25 compared UAA projects—the bullets indicate the features of the object in question. (compiled by A. Nowysz).

UAA OBJECT				UAA LOCATION															UAA PRODUCTION											
TYPE		No.	NAME (City)	Location in the City							Functional Zone					Space Type			Type of Producers			Aim of Production			Object of Production					
				Architectural Space			Urban Space				Housing Estate	School Area	Recreational Area	Multifunctional Area	Post-Industrial Area	Public	Semi-Public	Private	State	Non-State	Informal	Market	Self-Supply	Food Banks	Vegetables	Fruits	Cereal	Herbs	Decorative Plants	Animal Breeding
				Roof	Facade	Interior	Vacant lot	Street/Square	Green Area	Water Area																				
GARDENS	ALLOTMENT	1	Schrebergärten (Leipzig)				•						•				•			•	•		•		•	•		•	•	•
		2	ROD Wytchnienie (Wroclaw)						•		•		•			•				•	•		•		•	•		•	•	•
	COMMUNITY	3	Prinzessinengärten (Berlin)				•							•			•				•	•	•		•	•		•	•	•
		4	Allmende-Kontor (Berlin)				•		•				•			•					•		•		•	•		•	•	
		5	CarlsGarten (Cologne)					•							•		•				•		•		•	•		•	•	
		6	L’îlot Amaranthes (Lyon)				•	•			•					•				•	•		•		•	•		•	•	
		7	Ecobox (Paris)					•			•			•			•				•		•		•	•		•	•	
		8	Passage 56 (Paris)				•				•							•		•	•		•		•	•		•	•	
		9	Lafayette Greens (Detroit)				•							•			•			•	•		•	•	•	•		•	•	
		10	Edible School Yard 1 (New York)					•				•				•				•			•		•	•		•	•	
		11	Edible School Yard 2 (New York)					•				•				•				•			•		•	•		•	•	
FARMS	HORIZONTAL	12	Landgrab City (Shenzen)					•						•		•				•			•	•	•	•	•	•		
		13	Value Farm (Shenzen)					•							•	•				•			•	•	•	•		•		
		14	Urby Staten Island (New York)				•				•						•			•	•	•	•		•	•		•		•
		15	Houtan Park (Shanghai)						•				•			•				•			•	•	•	•	•	•		
		16	Floating Fields (Shenzen)					•		•					•	•				•			•	•	•	•		•		•
		17	Swale (New York)							•			•			•					•		•	•	•	•		•		
		18	Gary Comer YC (Chicago)	•										•				•		•	•	•	•		•	•		•		
		19	SYNTHe (Los Angeles)	•										•				•		•		•	•		•	•		•		
		20	La Sazon (Havana)				•				•							•	•	•		•	•	•	•	•	•	•		•
		21	MUFI (Detroit)				•				•							•			•		•	•	•	•		•		
		22	AgroCité (Paris)				•				•					•					•	•	•	•	•	•		•		•
	VERTICAL	23	Public Farm 1 (New York)					•						•			•			•		•	•	•	•	•		•		•
		24	American Food 2.0 (Milano)		•									•			•		•	•		•	•		•			•		
		25	Pasona Urban Farm (Tokio)	•	•	•								•				•		•			•		•	•	•	•		

## Supplementary Materials

The following supporting information can be downloaded at: https://www.mdpi.com/article/10.3390/ijerph192315597/s1, supplementary figures.
