# Supplementary material for: Urban Agriculture as an Alternative Source of Food and Water Security in Today’s Sustainable Cities
Source: Int J Environ Res Public Health. 2022 Nov 24;19(23):15597. doi: 10.3390/ijerph192315597 (PMC9739535; doi:10.3390/ijerph192315597)
Supplement: Supplementary file 1 [file ijerph-19-15597-s001.zip › ijerph-2020033-supplementary.pdf]

01	ALLOTMENT GARDEN	GERMANY
SCHREBERGÄRTEN		LIPZIG

1. GENERAL INFORMATION

- **Address:** Aachener Str. 7, 04109 Lipzig, Germany
- **Built in:** 1868
- **Total area (colony):** 26 200 m^2
- **Area (garden plot):** 125 m^2
- **Project (colony):** M. Schreber, H. Gessel
- **Project (garden plot):** user (tenant),
- **Financing (garden plot):** user (tenant)
- **Buil by (garden plot):** user (tenant)
- **Management:** Deutsches Kleingärtnermuseum
- **Project goal:** playground, gardens for families

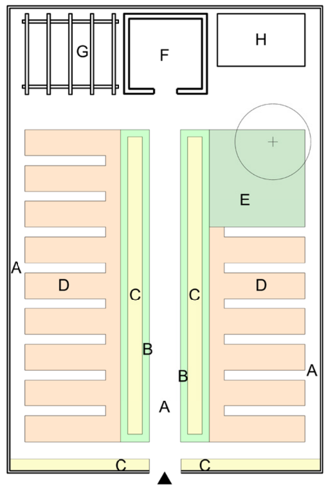

fig.S1 Scheme of a typical lot in the Schrebergärten garden colony in Leipzig [original study, based on local visit].

Explanations:
A – paths,
B – decoration hedge,
C – decoration flowers,
D – edible plants, E – fruit trees,
F – summerhouse,
G – terrace with construction for grapevine plants,
H – composter,

2. CONTEXT

- Garden colony in an undeveloped area
- Functional zone - recreation area
- Semi-public space

3. ARCHITECTURE

- Public square surrounded by garden plots
- Public, narrow alleys between plots
- Each plot has an arbor

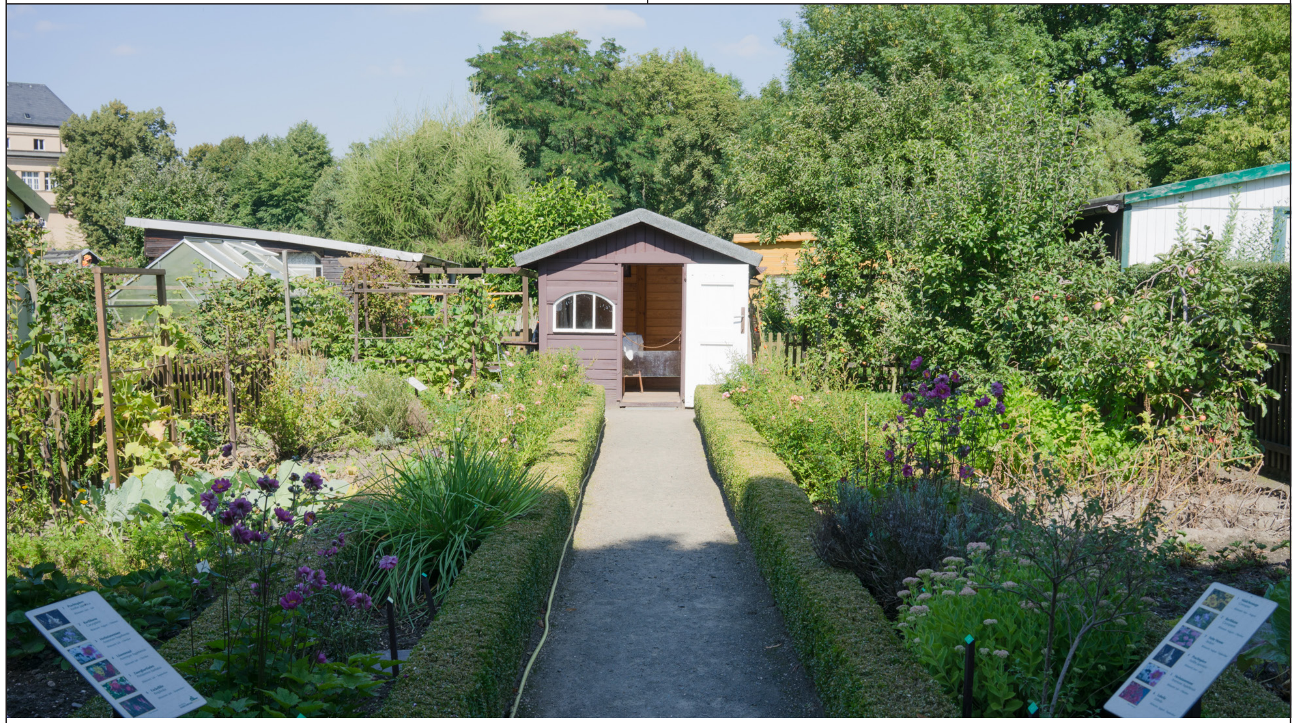

fig.S2 Schrebergärten garden colony in Leipzig - a typical lot (2017, original phot.).

4. FOOD PRODUCTION

- Purpose of production: self-supply
- Growing edible plants (herbs, fruits, vegetables)

5. OTHER FUNCTIONS

- Recreational and leisure function.
- Cultural function: Deutsches Kleingärtnermuseum

6. INFRASTRUCTURE AND TECHNOLOGY

- Soil-based
- Waste composting

7. PROJECT VALUE

- Ecological value: biologically active; water retention
- Functional use of green zones
- Social value: local place for the community; cultural spot; gardening education; broader access to fruit and vegetables

8. SOURCES

- Katsch G., Kosbi H., Kroß E., Leistner K. H., Philipp R., Uschpilkat E., Geschichte des Kleingartenwesens in Sachsen, Drezno 2007.
- www.kleingarten-museum.de/en/, (27.06.2017).
- www.google.pl/maps, (27.06.2017).
- local visit (15.07.2017).

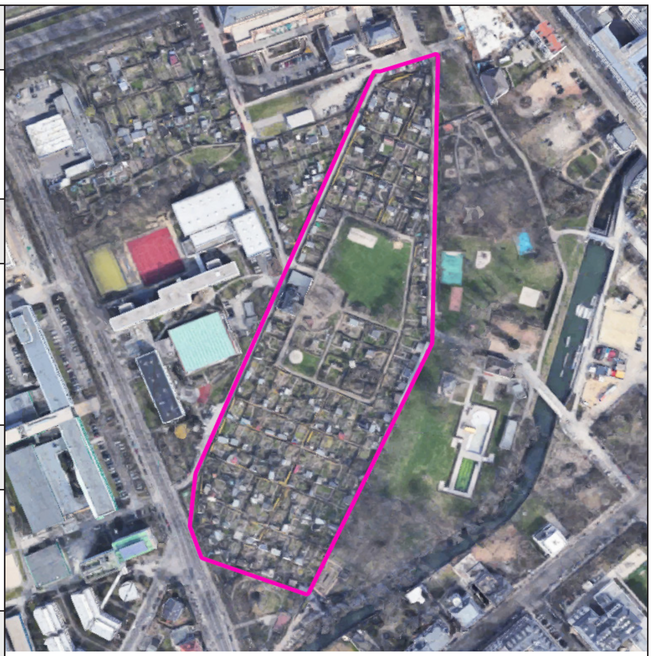

fig.S4 Location of the Schrebergärten garden colony in Leipzig [original study, based on: www.google.pl/maps, (27.06.2017)].

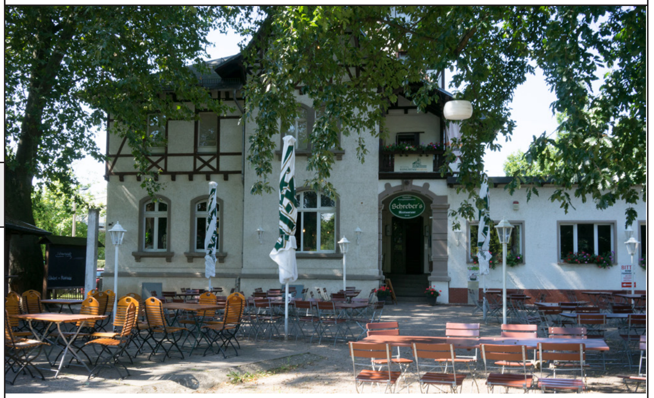

fig.S3 Schrebergärten garden colony in Leipzig - Deutsches Kleingärtnermuseum (2017, original phot.).

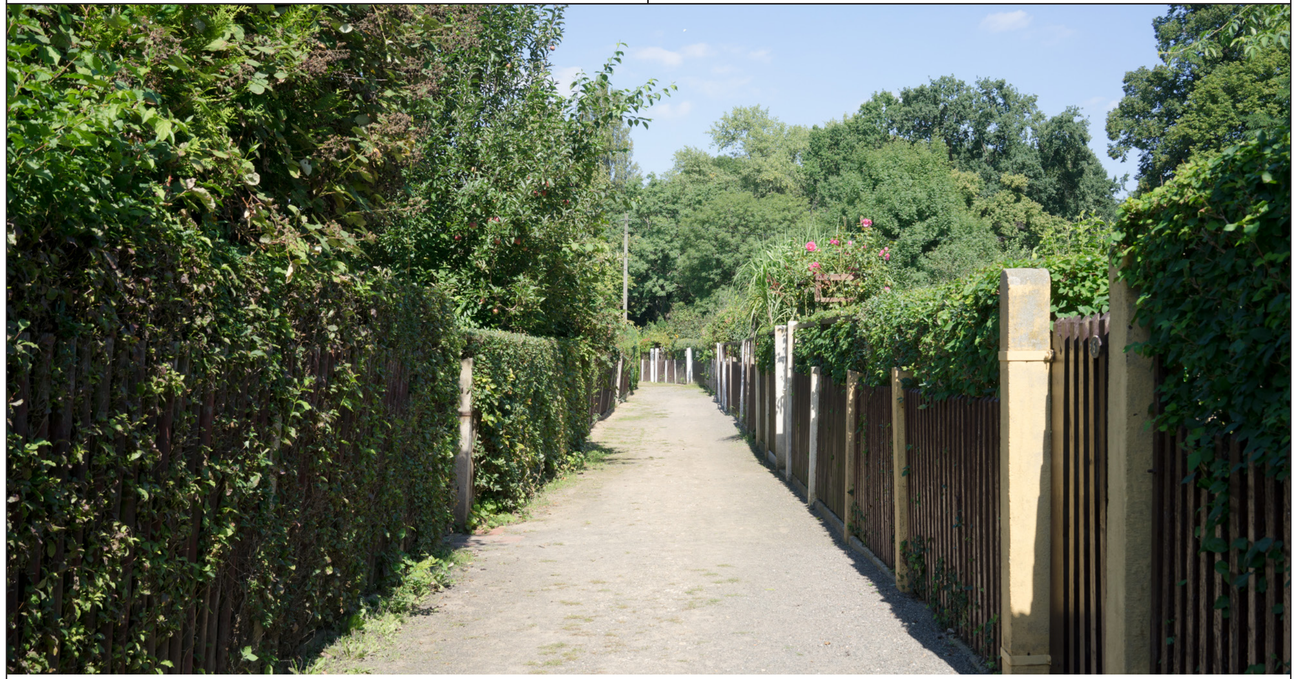

fig.S5 Schrebergärten garden colony in Leipzig - alley between plots (2017, original phot.).

02 ALLOTMENT GARDEN

POLAND

RODZINNY OGRÓD DZIAŁKOWY (ROD) WYTCHNIENIE

WROCŁAW

1. GENERAL INFORMATION

- **Address:** Kętrzyńska, 50-001 Wrocław, Poland
- **Built in:** 1930
- **Total area (colony):** 306 821 m^2
- **Area (garden plot):** 300 m^2
- **Project (colony):** T. Effenberger, H. Thomas
- **Project (garden plot):** użytkownicy,
- **Finansing (garden plot):** gardener (user)
- **Built by (garden plot):** gardener (user)
- **Management:** cooperative *ROD*, gardeners (users)
- **Project goal:** housing estate with gardens

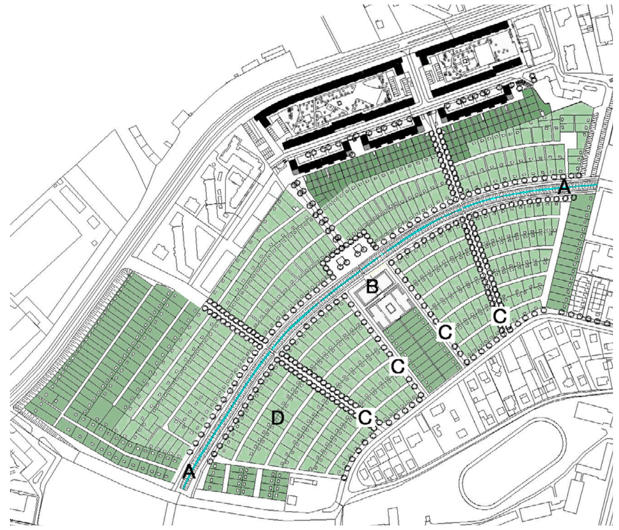

fig.S6 Scheme of the *ROD Wytchnienie* [original study, based on local visit and Ptaszycka A., 1956].

2. CONTEXT

- Garden housing estate
- Pulic park

Explanation:
A (blue) - public accessible walking alley with a canal in the middle;
B - central recreation area with a pool basin;
C - public accessible walking alleys;
D (green) - individual garden plots with arbors;

3. ARCHITECTURE

- Allotment gardens as part of a selfsufficient housing estate
- Garden-city idea
- Each plot has an arbor

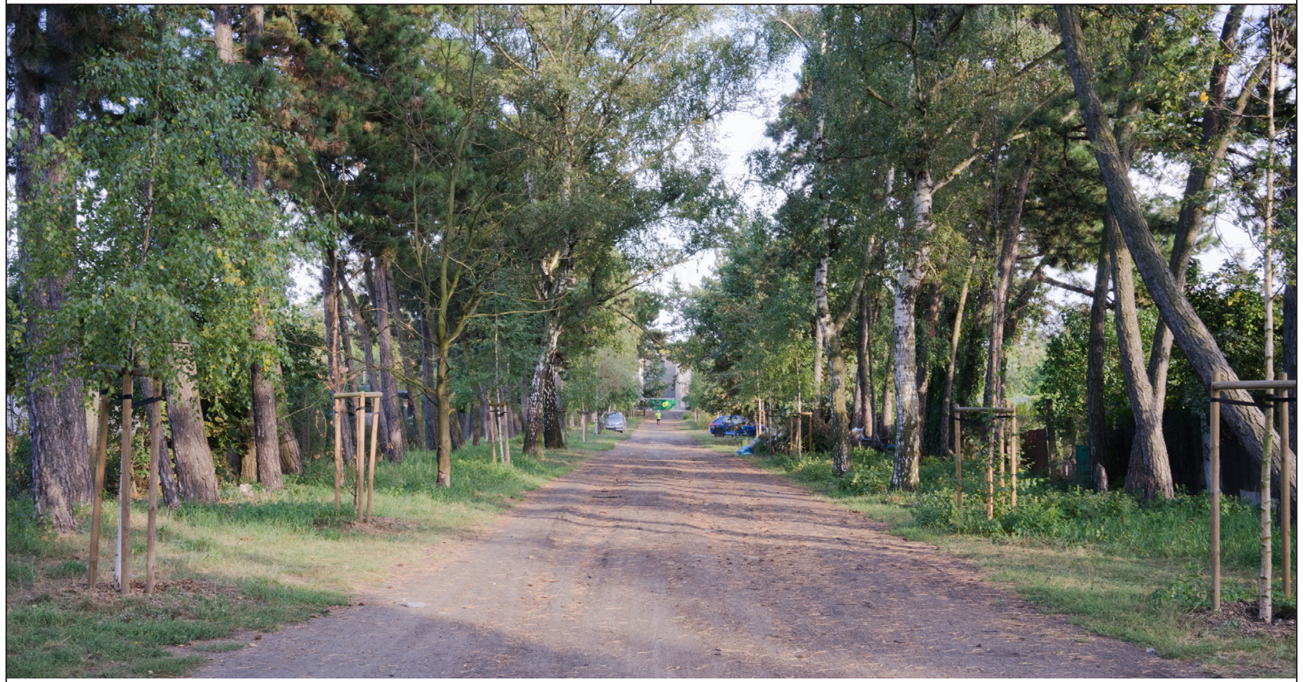

fig.S7 *ROD Wytchnienie* garden colony in Wrocław - alley between plots (2018, original phot.).

4. FOOD PRODUCTION

- Purpose of production: self-supply
- Growing edible plants (herbs, fruits, vegetables)

5. OTHER FUNCTIONS

- Recreational and leisure function

6. IINFRASTRUCTURE AND TECHNOLOGY

- Soil-based
- Waste composting

7. PROJECT VALUE

- Ecological value: biologically active; water retention
- Functional use of green zones
- Social value: local place for the community; cultural spot; gardening education; broader access to fruit and vegetables

8. SOURCES

- Decyzja w Sprawie Wpisania Zabytku do Rejestru Zabytków z dnia 12 grudnia 2008 r., Dolnośląski Wojewódzki Konserwator Zabytków we Wrocławiu, nr rejestru zabytków: A/1057.
- Maleczyński K., Morelowski M., Ptaszycka A., Wrocław. Rozwój urbanistyczny, Warszawa 1956, p. 307
- www.google.pl/maps, (27.06.2017)
- Local visit (20.08.2018).

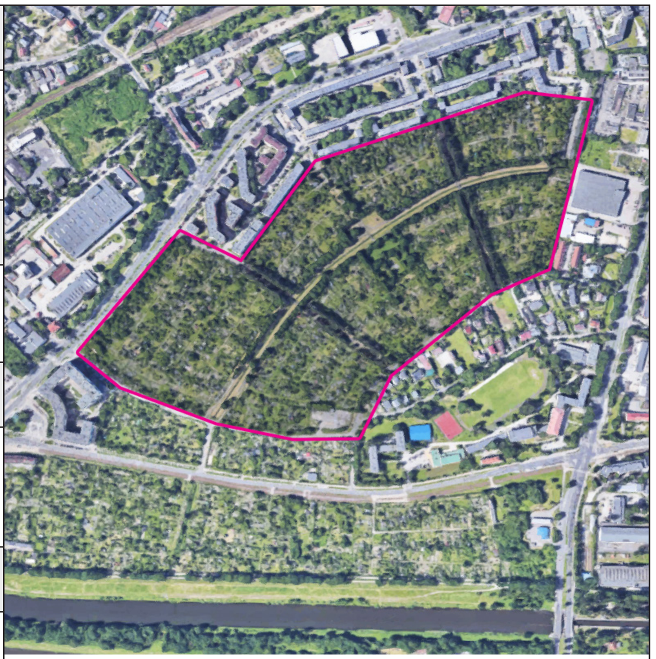

fig.S9 Location of the *ROD Wytchnienie* garden colony in Wrocław [original study, based on: www.google.pl/maps, (01.09.2018)].

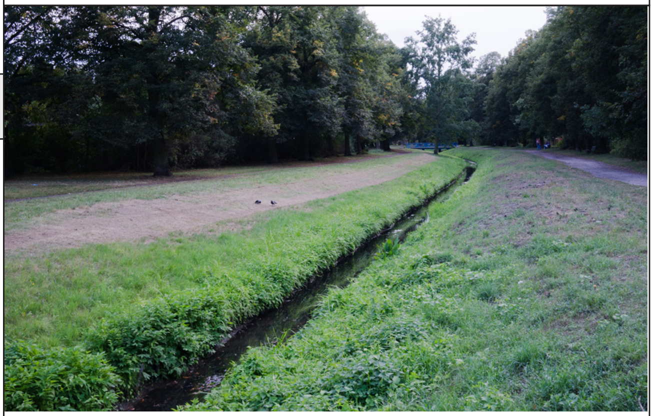

fig.S8 *ROD Wytchnienie* garden colony in Wrocław - alley with a canal in the middle (2018, original phot.).

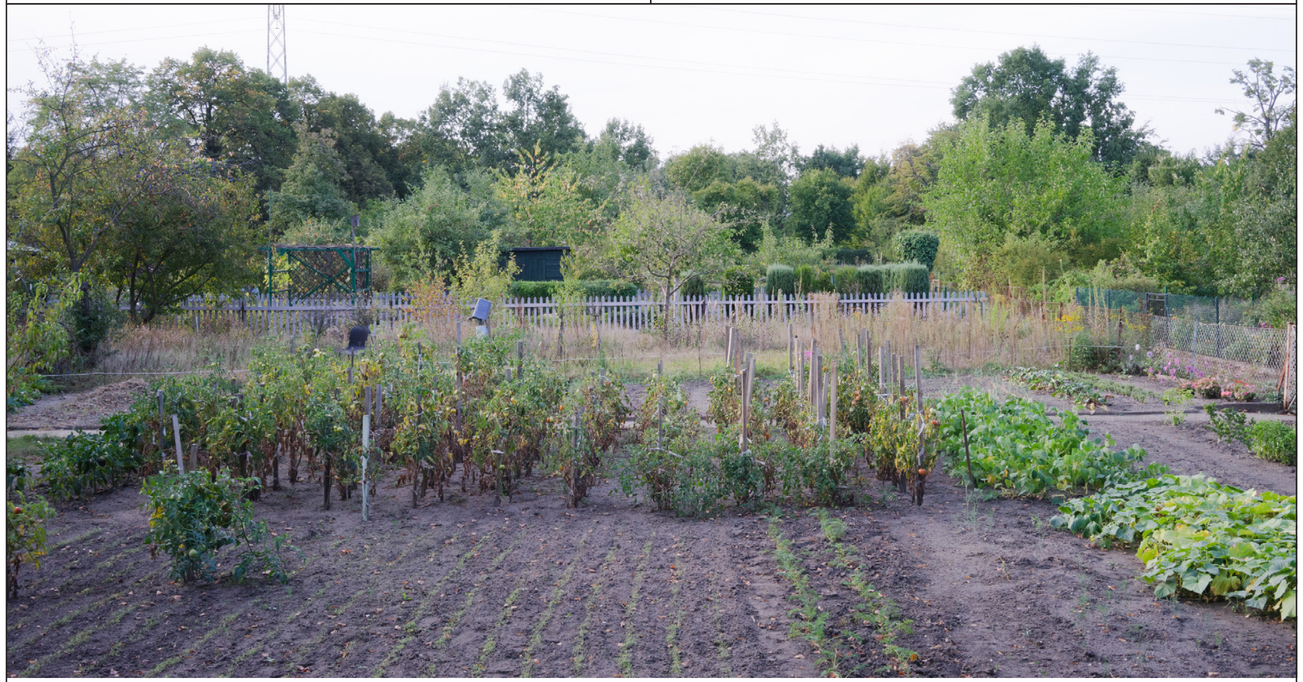

fig.S10 *ROD Wytchnienie* garden colony in Wrocław - individual plot (2019, original phot.).

03 COMMUNITY GARDEN — GERMANY

PRINZESSINNENGÄRTEN — BERLIN

1. GENERAL INFORMATION

- **Address:** Prinzenstraße 35-38, 10969 Berlin, Germany
- **Built in:** 2009
- **Total area:** 6 000 m^2
- **Project:** garden cooperative
- **Finansing:** garden cooperative, donations
- **Built by:** garden cooperative
- **Management:** garden cooperative
- **Project goal:** community garden

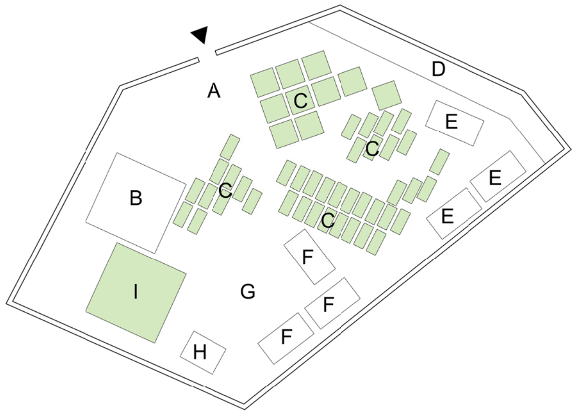

fig.S11 Scheme of the *Prinzessinnengärten* garden [original study, based on local visit].

Explanation:
A - entrance area,
B - stage,
C - mobile containers and installations for growing crops
D - multifunctional area
E - back room and toilets in containers
F - kitchen and bar in containers
G - gastronomic and recreational zone
H - apiary, I - garden nursery

2. CONTEXT

- Vacant lot in a city district
- Residential zone
- Semi-public space

3. ARCHITECTURE

- Garden established on a vacant lot
- Garden area divided on functional zones
- DIY garden containers and architecture

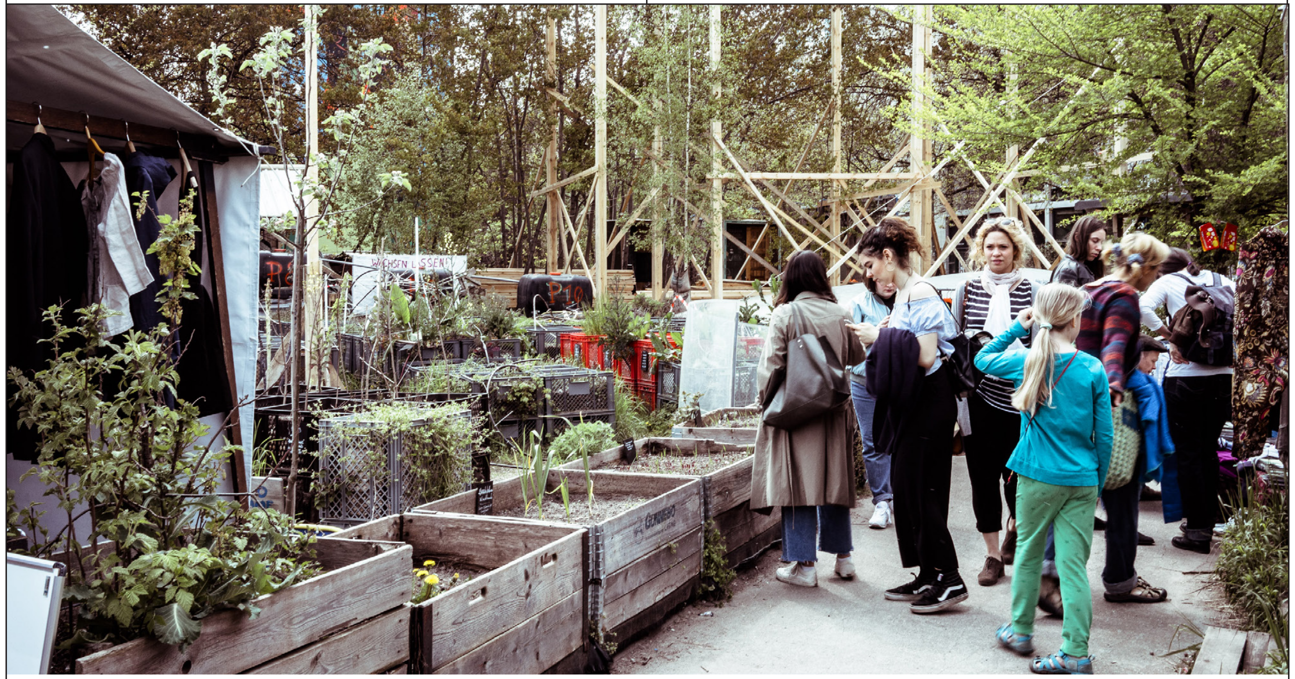

fig.S12 *Prinzessinnengärten* garden in Berlin - alley between plant beds (2016, original phot.).

4. FOOD PRODUCTION

- Purpose of production: self-supply, retail
- Growing edible plants (herbs, fruits, vegetables)
- Garden nursary
- Breeding: apiaries, chickens

5. OTHER FUNCTIONS

- Recreational and leisure function
- Cultural and educational function (events, workshops, lectures, exhibitions, film screenings, garden education, concerts, seminars).
- Retail trade: shop, weekend market.
- Gastronomy: bistro.

6. IINFRASTRUCTURE AND TECHNOLOGY

- Soil-based
- Waste composting and rainwater container

7. PROJECT VALUE

- Urban renewal of a vacant lot
- Semipublic space - open daily at a certain time
- Social value: local place for the community; cultural spot; gardening education; broader access to fruit and vegetables

8. SOURCES

- www.prinzessinnengarten.net/about/, (09.06.2016); www.google.pl/maps, (27.06.2017).
- Local visit (01.05.2016).

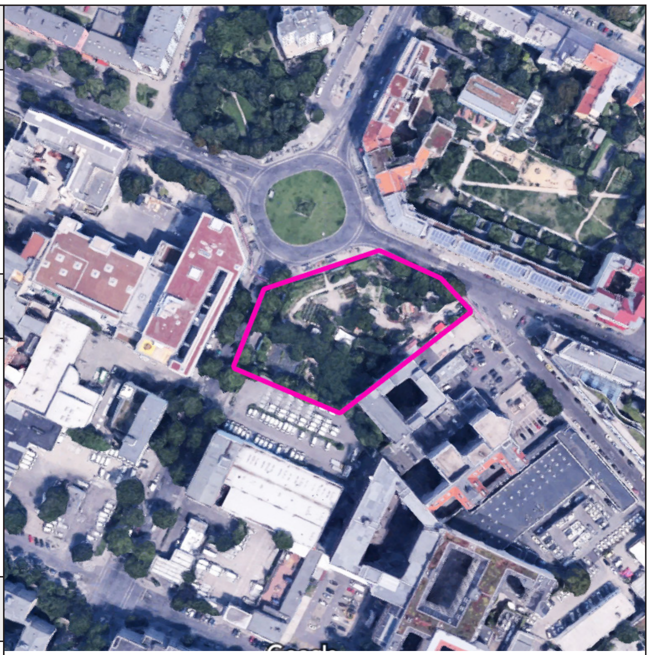

fig.S14 Location of the P*rinzessinnengärten* garden in Berlin [original study, based on: www.google.pl/maps, (01.09.2018)].

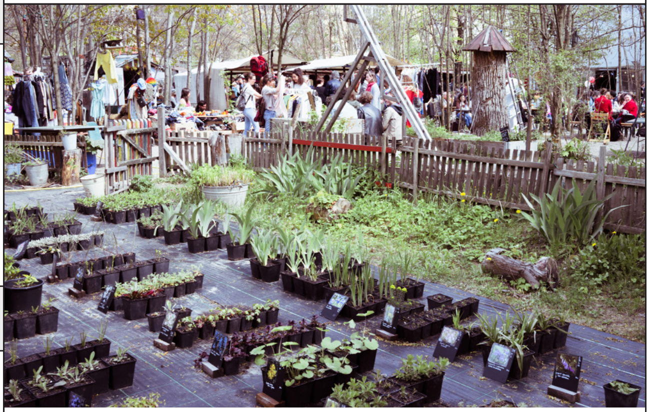

fig.S13 *Prinzessinnengärten* garden in Berlin - a garden nursary (2016, original phot.).

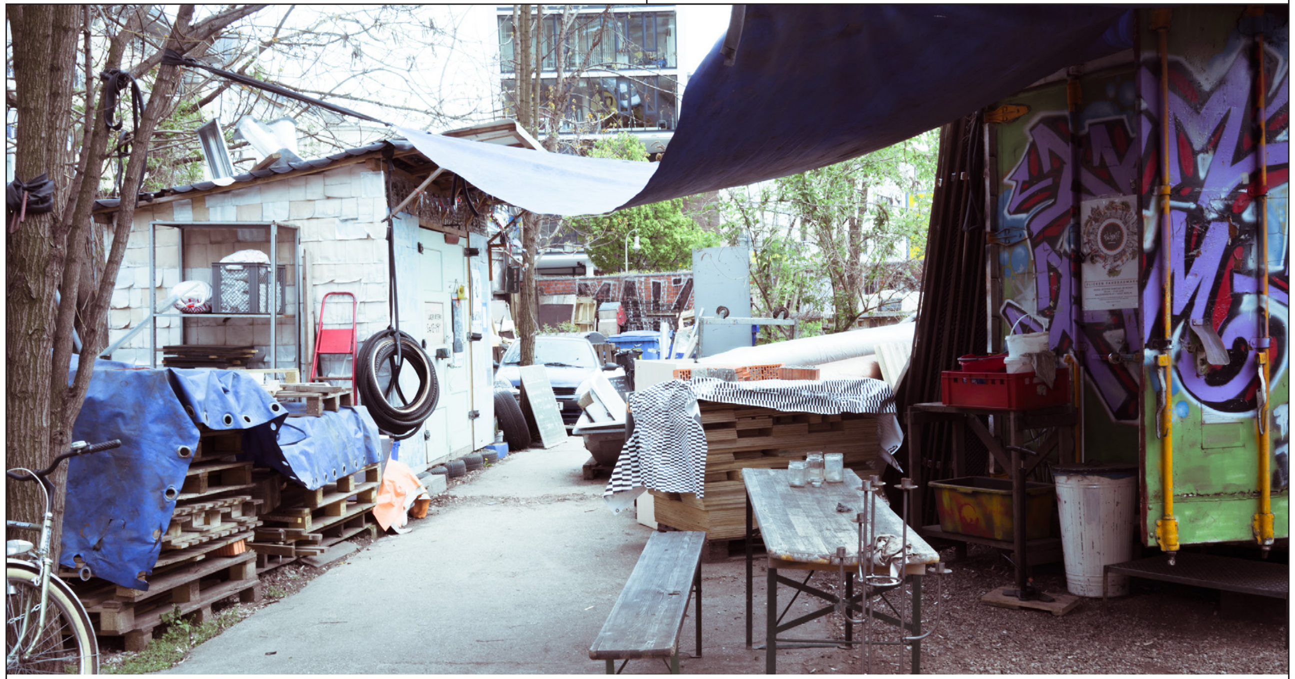

fig.S15 *Prinzessinnengärten* garden in Berlin - alley between „anarchitecture" objects (2016, original phot.).

04 COMMUNITY GARDEN — GERMANY

ALLMENDE-KONTOR — BERLIN

1. GENERAL INFORMATION

- **Address:** Oderstrasse, Tempehofer Feld, Berlin, Germany
- **Built in:** 2011
- **Area:** approx 8 000 m^2
- **Project:** gardeners (users)
- **Finansing:** gardeners (users)
- **Buil by:** użytkownicy
- **Management:** garden cooperative, individualgardeners (users)
- **Project goal:** food production, leisure place

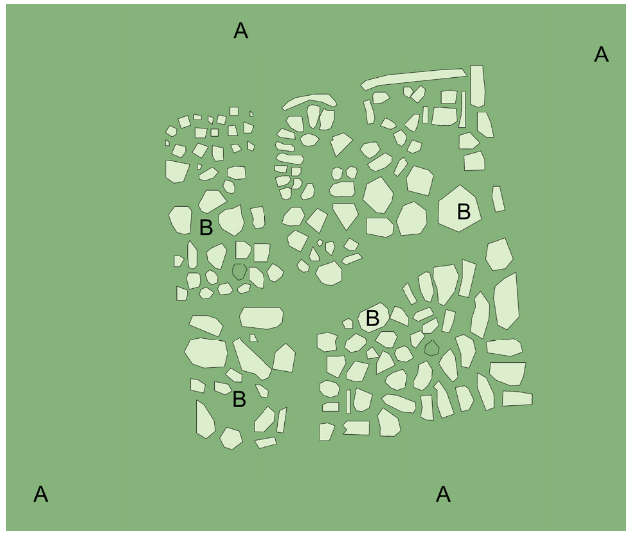

fig.S16 Scheme of the *Allmende-Kontor* garden [original study, based on local visit].

Explanation:
A - public park (former airport area)
B - *Allmende-Kontor* garden - DIY beds and installations for growing crops

2. CONTEXT

- Former airport
- Recreational area
- Public space

3. ARCHITECTURE

- Open-plan community garden: garden containers and beds - freely positioned in the public space.
- DIY objects

4. FOOD PRODUCTION

- Purpose of production: self-supply
- Growing edible plants (herbs, fruits, vegetables)
- Apiaries

5. OTHER FUNCTIONS

- Recreational and leisure function
- Public space

6. INFRASTRUCTURE AND TECHNOLOGY

- Soil-based
- Waste composting
- Rainwater container

7. PROJECT VALUE

- Urban renewal of a vacant lot
- Public space
- Social value: local place for the community; cultural spot; broader access to fruit and vegetables

8. SOURCES

- www.allmende-kontor.de/index.php/gemeinschaftsgarten.html, (24.01.2018).
- www.google.pl/maps, (27.06.2017).
- Local visit (02.05.2016).

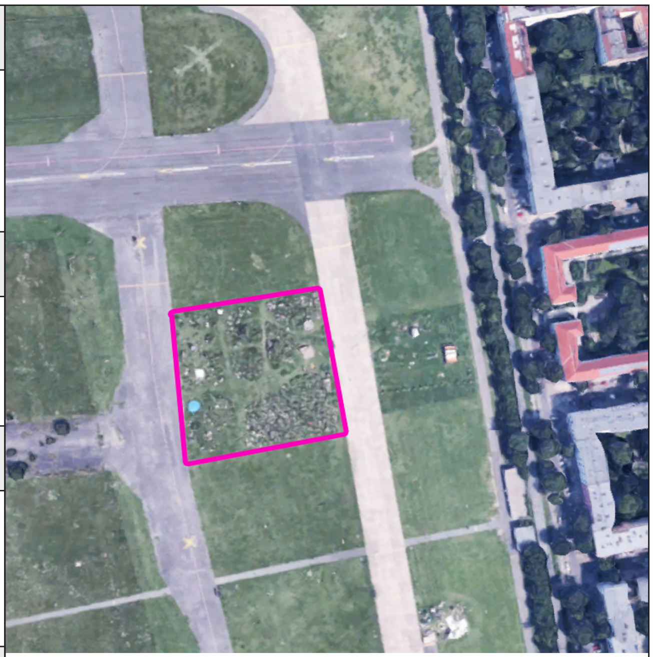

fig.S19 Location of the *Allmende-Kontor* garden in Berlin [original study, based on: www.google.pl/maps, (01.09.2018)].

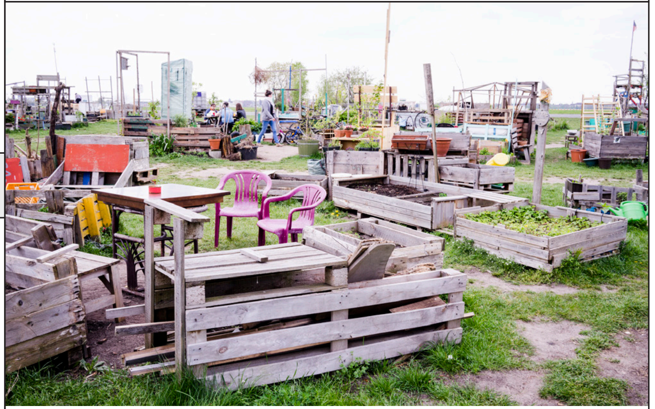

fig.S18 *Allmende-Kontor* garden in Berlin - plant beds (2016, original phot.).

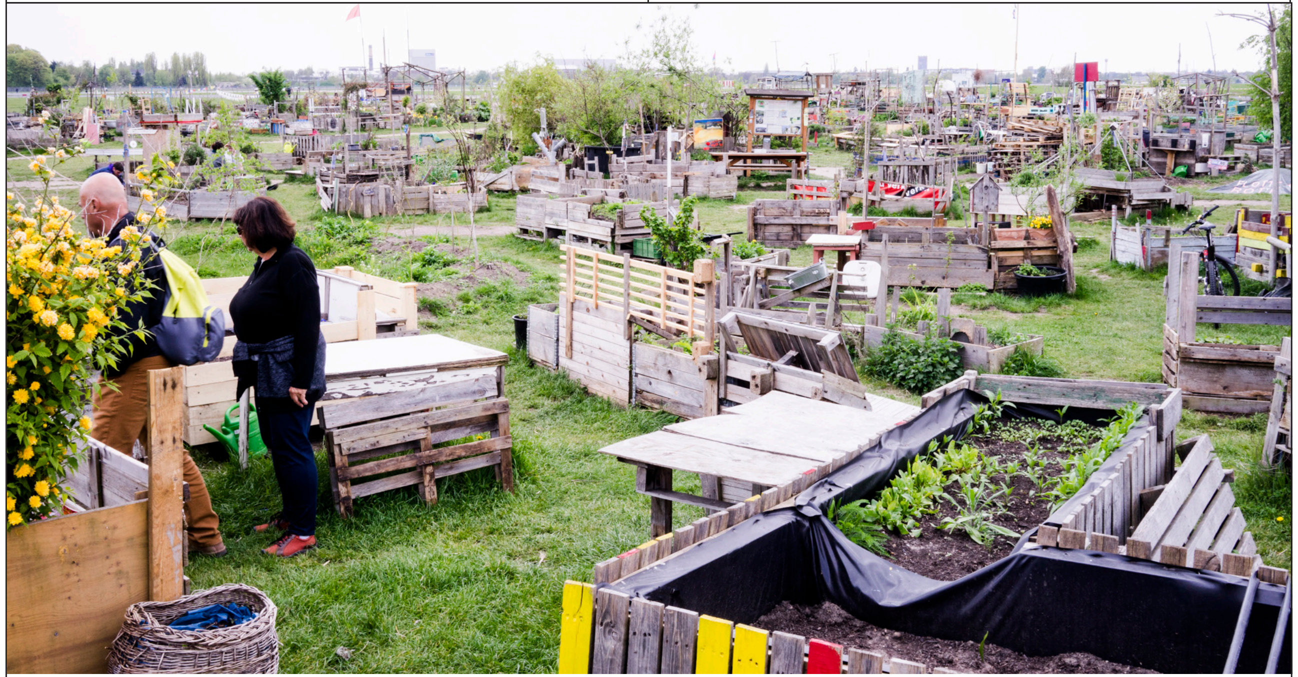

fig.S17 *Allmende-Kontor* garden in Berlin - plant beds (2016, original phot.).

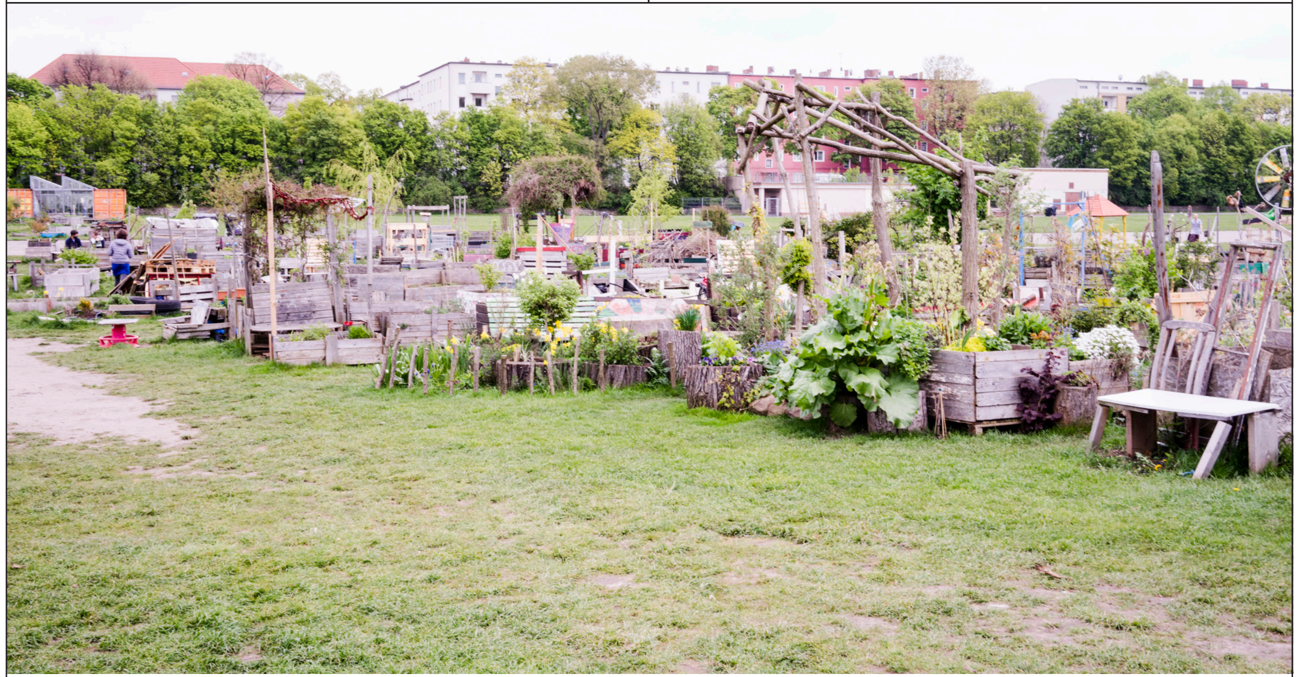

fig.S20 *Allmende-Kontor* garden in Berlin - a garden containers in the former Tempelhof Airport (2016, original phot.).

05 COMMUNITY GARDEN — FRANCE

ECOBOX — PARIS

1. GENERAL INFORMATION

- **Address:** 7 Impasse de la Chapelle, Paris, France
- **Built in (1st garden):** 2001
- **Demolished (1st garden):** 2005
- **Built in (3rd garden):** 2008
- **Total area (3rd garden):** 750 m^2
- **Project:** Atelier d'Architecture Autogérée (AAA) , gardeners (users)
- **Financing:** Municipality
- **Bulit by:** Atlier AAA, gardeners (users)
- **Management:** garden cooperative
- **Project goal:** community garden

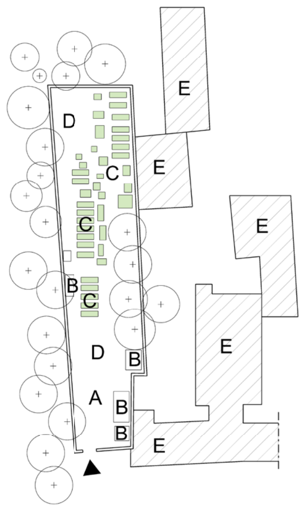

fig.S21 Scheme of the Ecobox garden [original study, based on local visit].

Explanations:
A – entrance zone
B – arbors
C – plants beds
D – leisure zone
E – residential buildings
F – railway zone

2. CONTEXT

- Plot between residential buildings and the railway infrastructure area
- Downtown
- Semi-public space

3. ARCHITECTURE

- Garden established on a vacant lot
- DIY garden containers and leisure architecture

4. FOOD PRODUCTION

- Purpose of production: self-supply
- Growing edible plants (herbs, fruits, vegetables)

5. OTHER FUNCTIONS

- Recreational and leisure function.

6. INFRASTRUCTURE AND TECHNOLOGY

- Soil-based
- Waste composting

7. PROJECT VALUE

- Urban renewal of a vacant lot
- Social value: local place for the community; cultural spot; gardening education; broader access to fruit and vegetables, participatory design

8. SOURCES

- www.urbantactics.org/wp-content/uploads/2015/09/portfolio-web-2015.pdf, (01.02.2018).
- www.ryerson.ca/carrotcity/board_pages/community/ecobox.html, (01.02.2018).
- www.google.pl/maps, (27.06.2017).
- Local visit (22.07.2016).

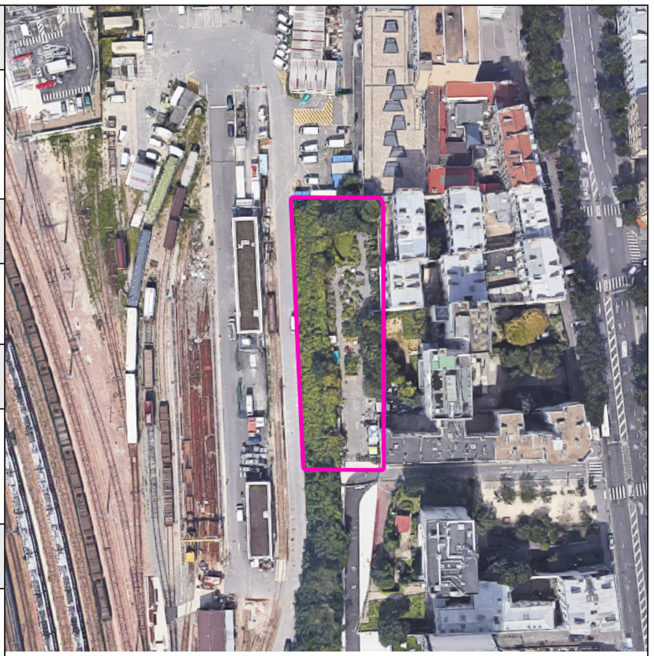

fig.S24 Location of the Ecobox garden in Paris [original study, based on: www.google.pl/maps, (22.07.2016)].

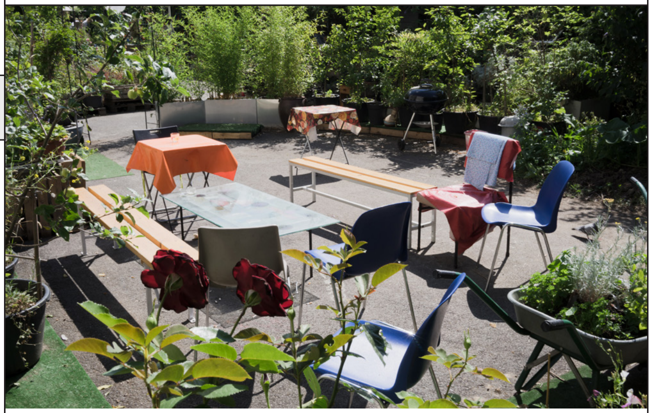

fig.S23 Ecobox garden - leisure zone (2016, original phot.).

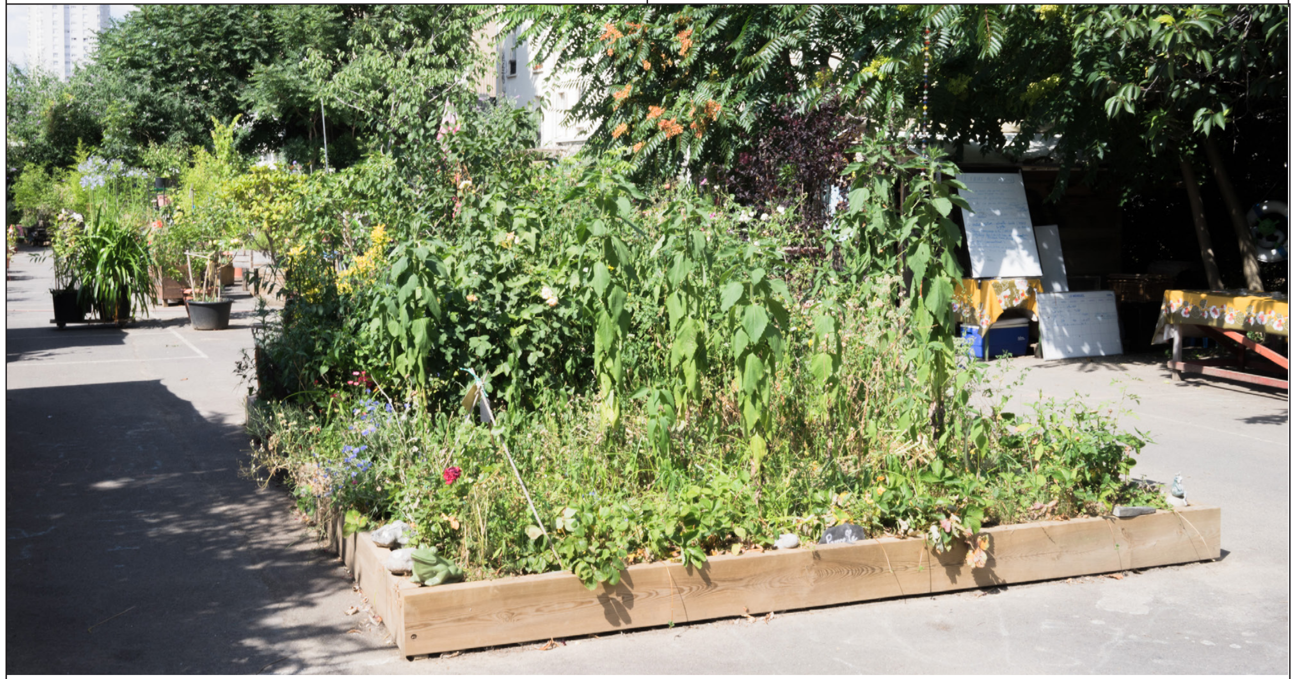

fig.S22 Ecobox garden - plant bed (2016, original phot.).

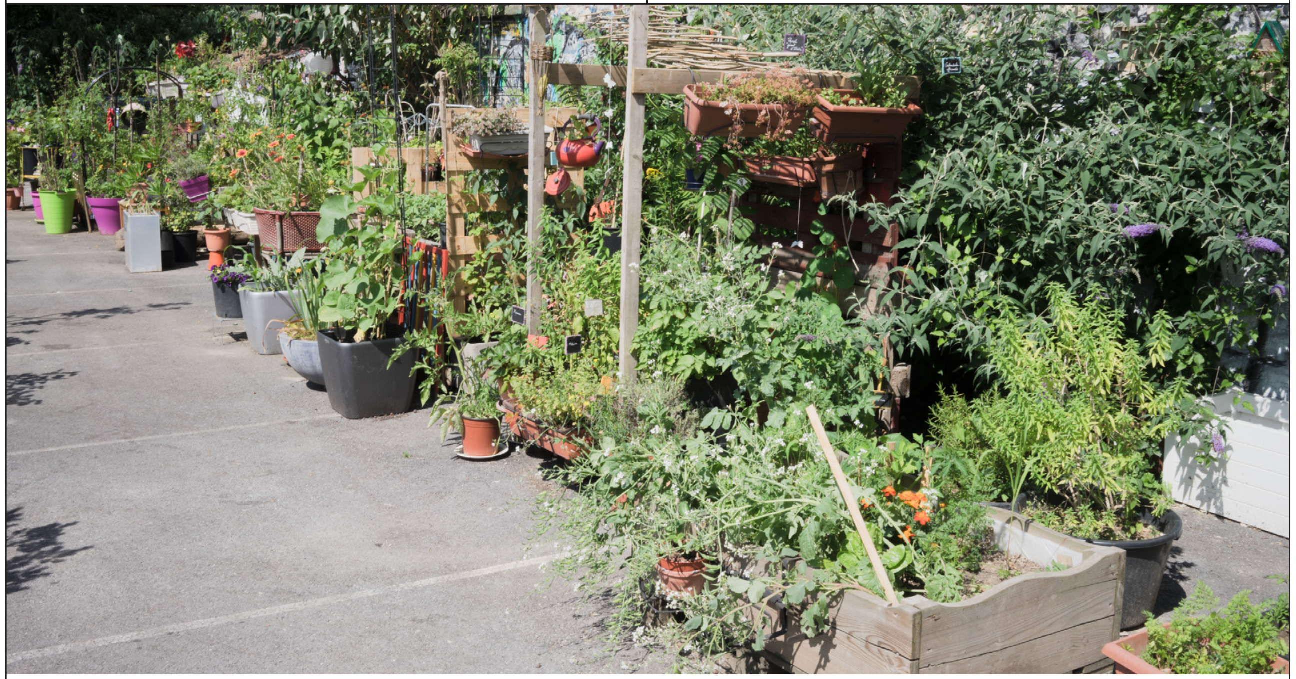

fig.S25 Ecobox garden - plant beds along an alley (2016, original phot.).

06 COMMUNITY GARDEN — FRANCE

PASSAGE 56 — PARIS

1. GENERAL INFORMATION

- **Address:** Rue Saint-Blaise 56, 75020 Paris, France
- **Buil in:** 2009
- **Total area:** 200 m^2
- **Project:** Atelier d'Architecture Autogérée (AAA), gardeners (users)
- **Finansing:** Municipality
- **Built by:** gardeners (users), volunteers
- **Management:** garden cooperative
- **Project goal:** community garden

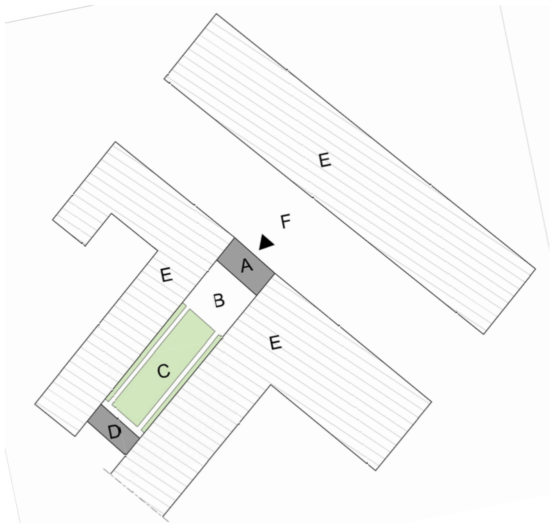

fig.S26 Scheme of the *Passage 56* garden [original study, based on local visit].

Explanation:
A - pavilion with an entrance gate
B - recreation area
C - crops zone
D -back room and composter
E - residential buildings
F - pedestrian and bicycle boulevard

2. CONTEXT

- Vacant lot in a narrow passage between residential buildings
- Residential estate
- Private space

3. ARCHITECTURE

- Garden established in a narrow passage
- Garden area divided on functional zones
- Entrance pavilion

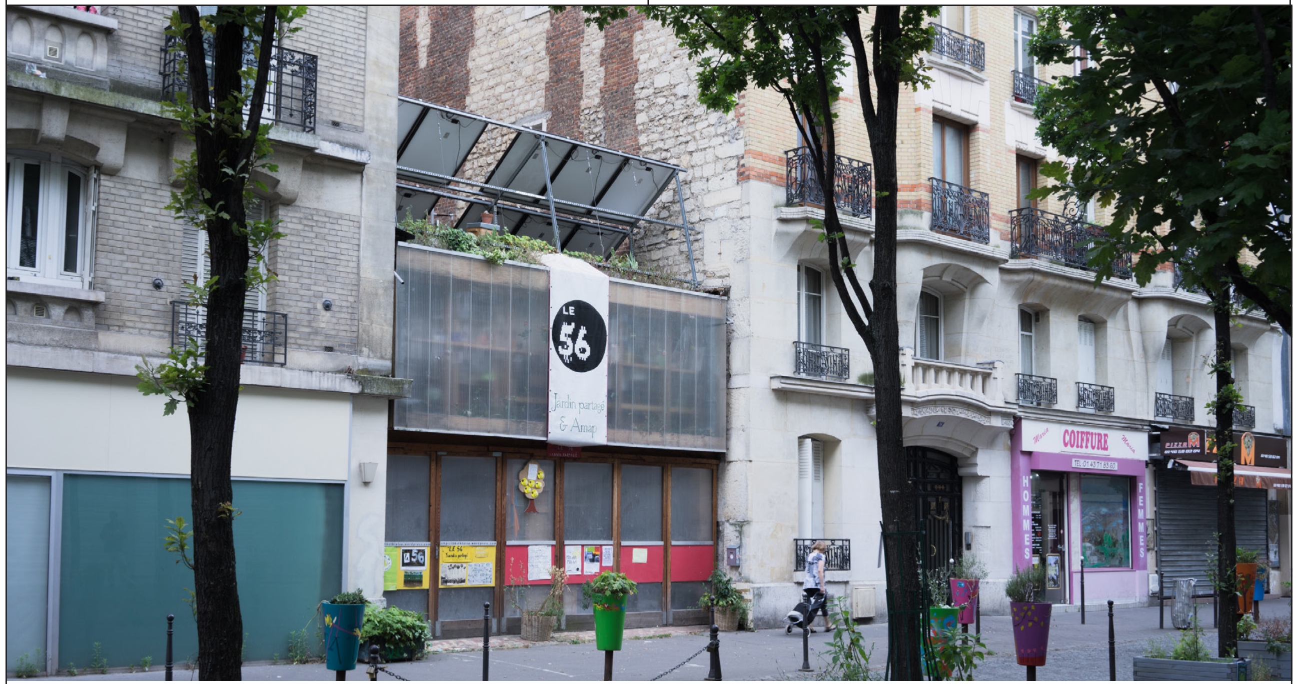

fig.S27 A community garden *Passage 56* (Paris) – entrance (2016, original phot.).

4. FOOD PRODUCTION

- Purpose of production: self-supply, retail
- Growing edible plants (herbs, fruits, vegetables)

5. OTHER FUNCTIONS

- Recreational and leisure function
- Cultural and educational function (events, workshops, lectures, exhibitions, film screenings, garden education, concerts, seminars).
- Retail trade

6. INFRASTRUCTURE AND TECHNOLOGY

- Soil-based
- Waste composting
- Rainwater container
- Solar panels

7. PROJECT VALUE

- Urban renewal of a vacant lot
- Social value: local place for the community; cultural spot; broader access to fruit and vegetables

8. SOURCES

- www.publicspace.org/en/works/f250-passage-56-espace-culturel-ecologique/prize:2010, (31.01.2018).
- www.56stblaise.wordpress.com/18/, (31.01.2018).
- www.urbantactics.org/projets/passage56/, (31.01.2018)
- www.google.pl/maps, (27.06.2017).
- Local visit (21.07.2016).

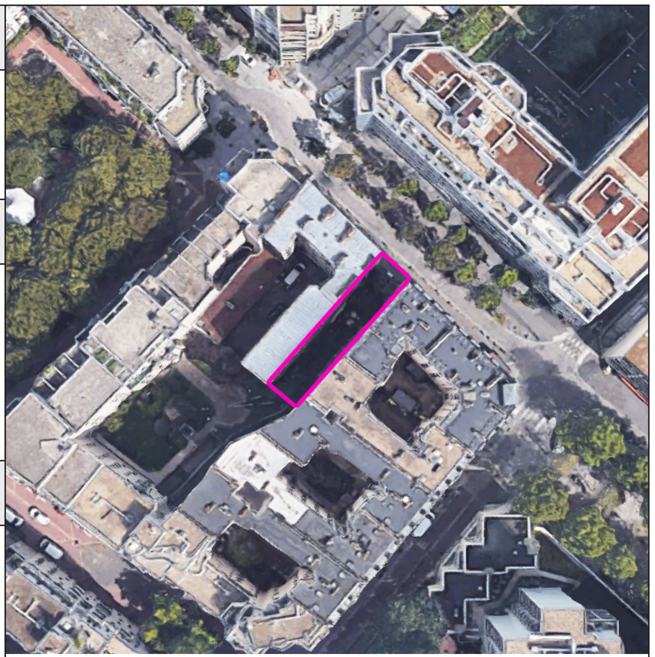

fig.S29 Location of the *Passage 56* garden in Berlin [original study, based on: www.google.pl/maps, (01.09.2018)].

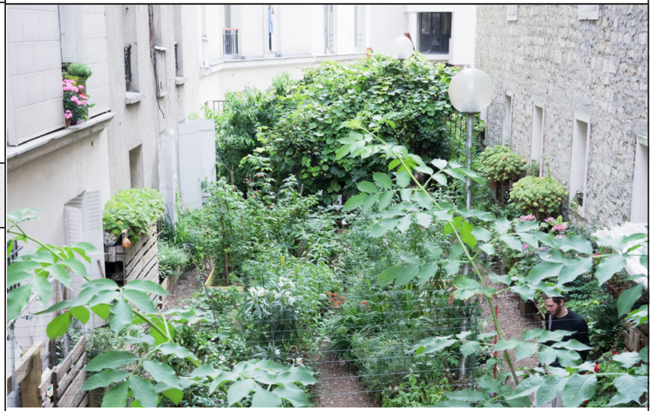

fig.S28 A community garden *Passage 56* (Paris) – a garden plot between two buildings (2016, original phot.).

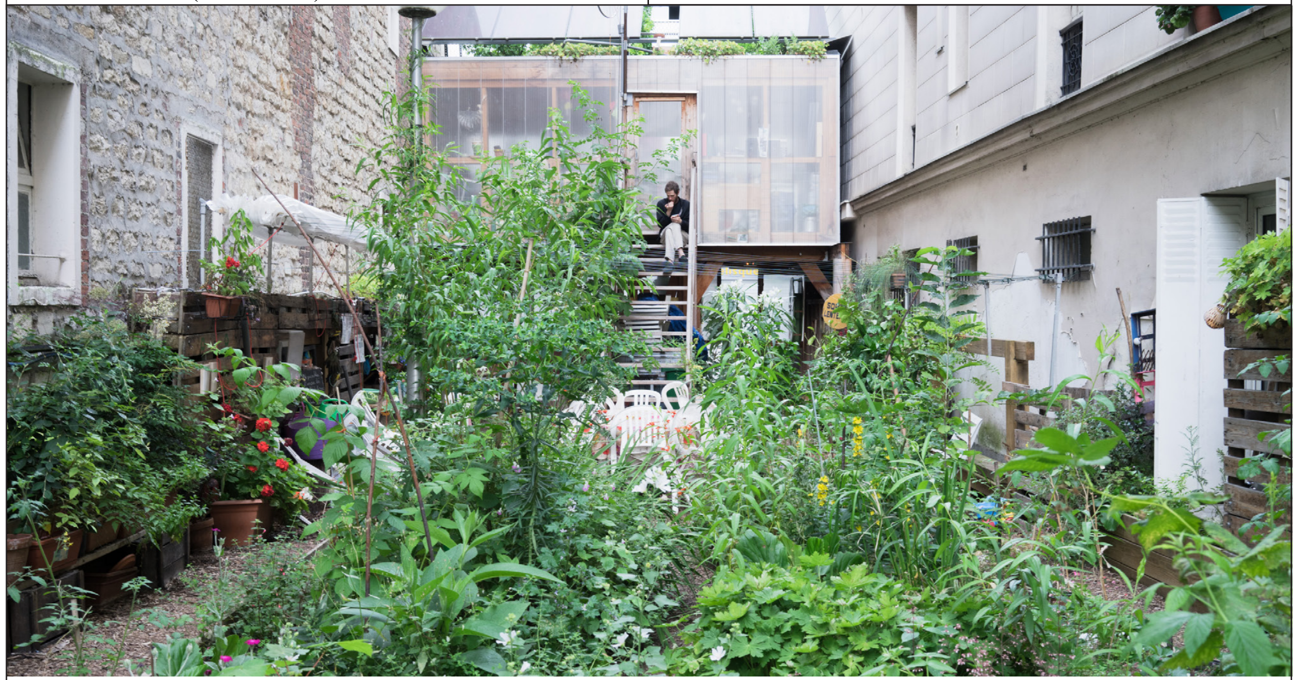

fig.S30 A community garden *Passage 56* (Paris) – a garden plot between two residential buildings (2016, original phot.).

07	URBAN FARM	FRANCE
AGROCITÉ		PARIS

1. GENERAL INFORMATION

- **Address:** J. Michelet 4-12, Colombos, Paris, France
- **Built in:** 2010–2014
- **Total area:** 2 800 m²
- **Crops area:** 900 m²
- **Project:** Atelier d’Architecture Autogérée (AAA)
- **Finansing:** Municipality
- **Buil by:** Atelier AAA, gardeners (users)
- **Management:** garden cooperative
- **Project goal:** community spot, foodhub
- **New location since:** 2018

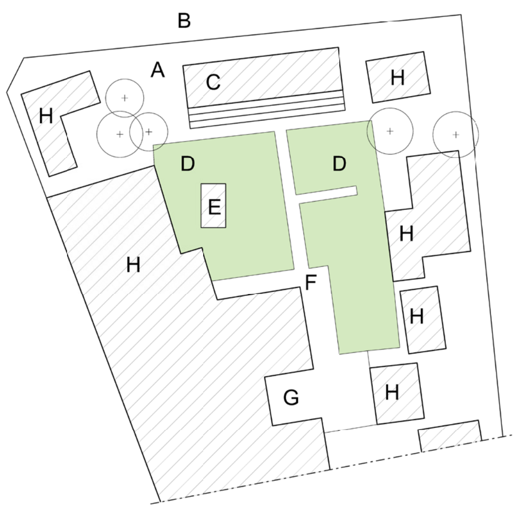

fig.S31 Scheme of the Agrocité farm [original study, based on local visit]

Explanation:
A – entrace zone
B – street
C – pavilion
D – crops area
E – foil tunnel
F – path
G – utility room
H – residential building

2. CONTEXT

- Vacant lot on suburbia
- Residential zone
- Semi-public space

3. ARCHITECTURE

- Urban field
- A wooden structure pavilion with collective kitchen

4. FOOD PRODUCTION

- Purpose of production: self-supply, retail
- Growing edible plants (herbs, fruits, vegetables)
- Seed library and garden nursery
- Breeding chickens, apiary

5. OTHER FUNCTIONS

- Gastronomy: cafe, collective kitchen

6. INFRASTRUCTURE AND TECHNOLOGY

- Soil-based and soilless
- Waste composting
- Solar panels
- Rainwater container
- Foil tunnel

7. PROJECT VALUE

- Urban renewal of a vacant lot
- Social value: local place for the community; cultural spot; gardening education; broader access to fruit and vegetables, local foodhub

8. SOURCES

- www.r-urban.net/, (31.01.2018).
- www.urbantactics.org/projets/agrocite/, (31.01.2018).
- www.google.pl/maps, (27.06.2017).
- Local visit (21.07.2016).

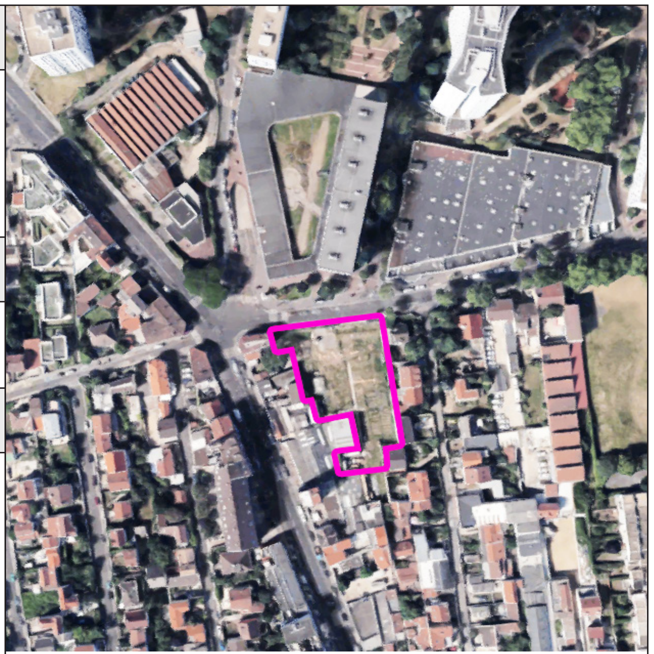

fig.S34 Exlocation of the Agrocité in Paris [original study, based on: www.google.pl/maps, (21.07.2016)].

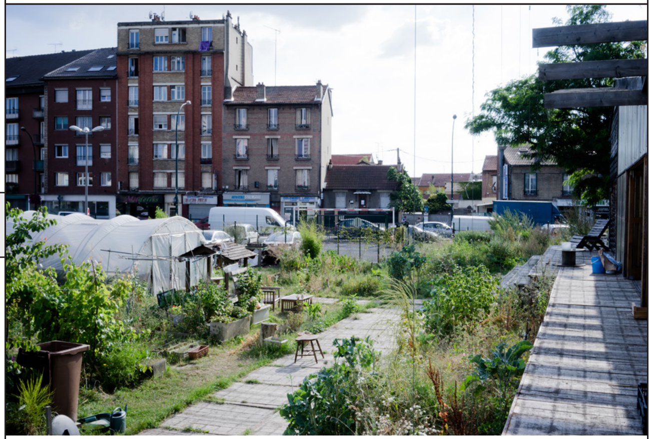

fig.S33 Agrocité farm (2016, original phot.).

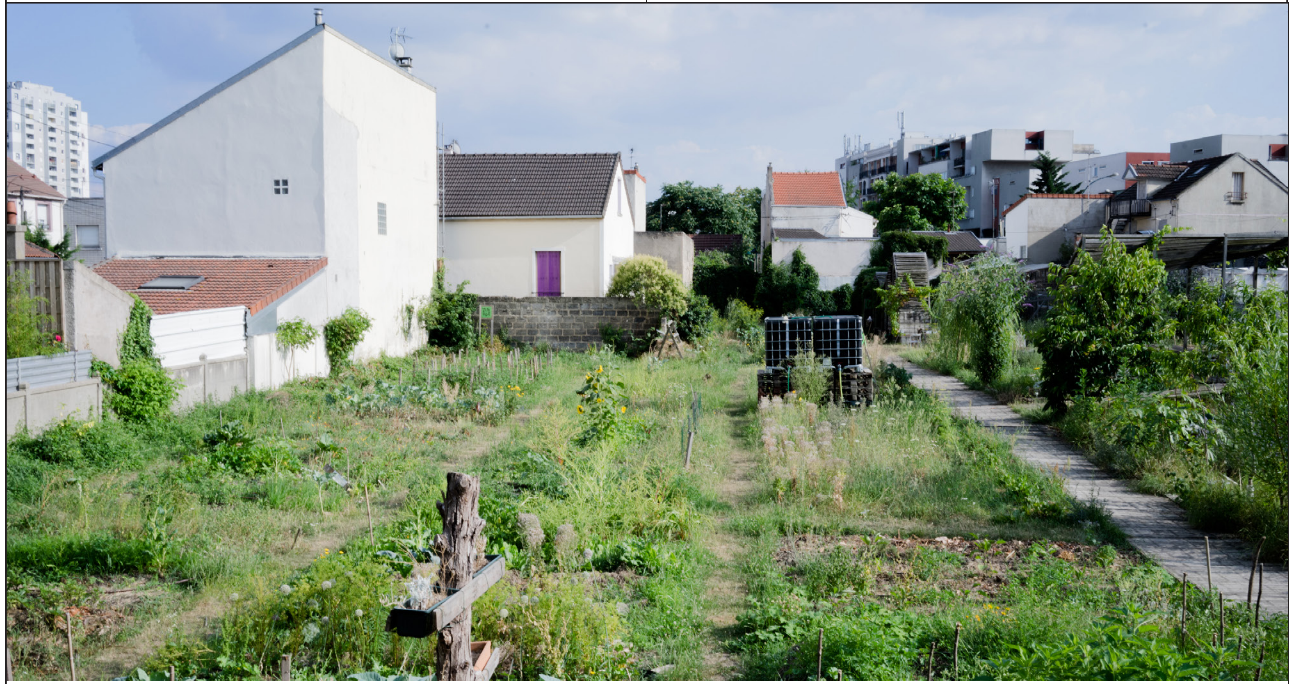

fig.S32 Agrocité farm - urban field (2016, original phot.).

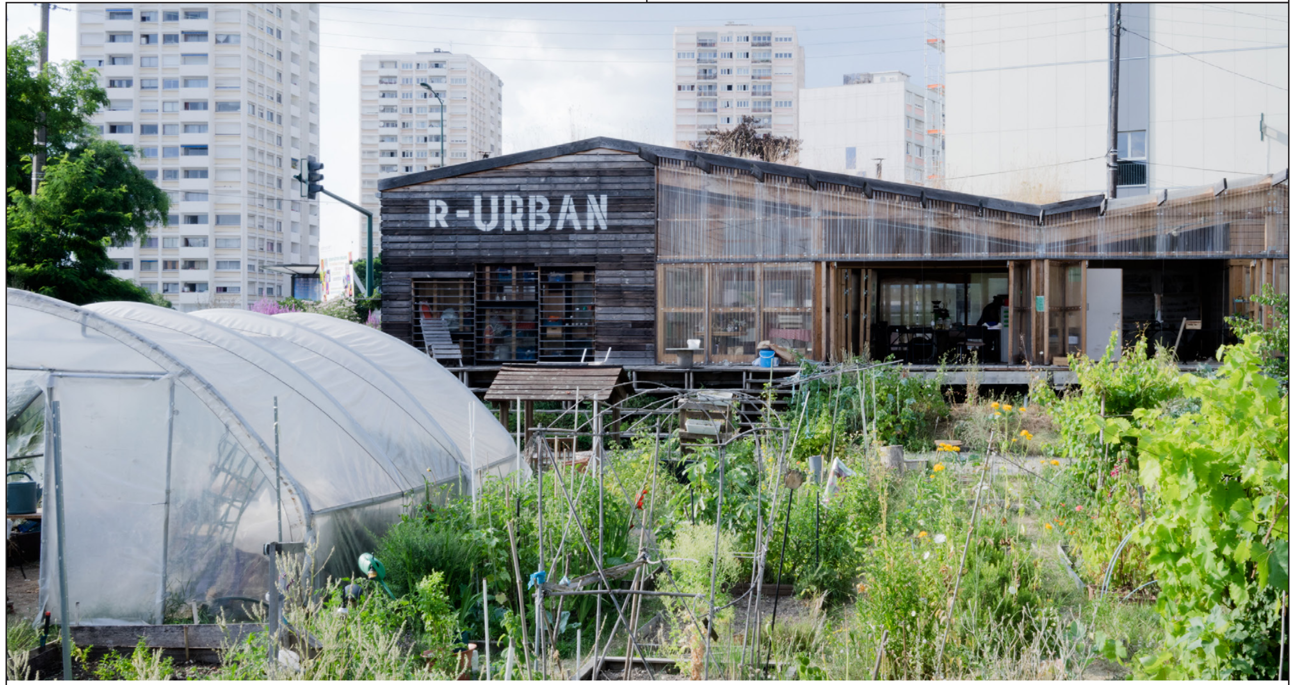

fig.S35 Agrocité farm - a pavilion (2016, original phot.).
